# Rescue of neurogenesis and age‐associated cognitive decline in SAMP8 mouse: Role of transforming growth factor‐alpha

**DOI:** 10.1111/acel.13829

**Published:** 2023-05-12

**Authors:** Ricardo Gómez‐Oliva, Sergio Martínez‐Ortega, Isabel Atienza‐Navarro, Samuel Domínguez‐García, Carlos Bernal‐Utrera, Noelia Geribaldi‐Doldán, Cristina Verástegui, Abdellah Ezzanad, Rosario Hernández‐Galán, Pedro Nunez‐Abades, Monica Garcia‐Alloza, Carmen Castro

**Affiliations:** ^1^ Área de Fisiología, Facultad de Medicina Universidad de Cádiz Cádiz Spain; ^2^ Instituto de Investigación e Innovación Biomédica de Cádiz (INIBICA) Cádiz Spain; ^3^ Departamento de Fisioterapia Universidad de Sevilla Seville Spain; ^4^ Departamento de Anatomía y Embriología Humanas, Facultad de Medicina Universidad de Cádiz Cádiz Spain; ^5^ Departamento de Química Orgánica Universidad de Cádiz Puerto Real Spain; ^6^ Departamento de Fisiología Universidad de Sevilla Sevilla Spain; ^7^ Present address: Department of Neuroscience Karolinska Institutet, Biomedicum Stockholm Sweden

**Keywords:** adult hippocampal neurogenesis, aging, dentate gyrus, diterpenes, memory, neuroregeneration, transforming growth factor‐alpha

## Abstract

Neuropathological aging is associated with memory impairment and cognitive decline, affecting several brain areas including the neurogenic niche of the dentate gyrus of the hippocampus (DG). In the healthy brain, homeostatic mechanisms regulate neurogenesis within the DG to facilitate the continuous generation of neurons from neural stem cells (NSC). Nevertheless, aging reduces the number of activated neural stem cells and diminishes the number of newly generated neurons. Strategies that promote neurogenesis in the DG may improve cognitive performance in the elderly resulting in the development of treatments to prevent the progression of neurological disorders in the aged population. Our work is aimed at discovering targeting molecules to be used in the design of pharmacological agents that prevent the neurological effects of brain aging. We study the effect of age on hippocampal neurogenesis using the SAMP8 mouse as a model of neuropathological aging. We show that in 6‐month‐old SAMP8 mice, episodic and spatial memory are impaired; concomitantly, the generation of neuroblasts and neurons is reduced and the generation of astrocytes is increased in this model. The novelty of our work resides in the fact that treatment of SAMP8 mice with a transforming growth factor‐alpha (TGFα) targeting molecule prevents the observed defects, positively regulating neurogenesis and improving cognitive performance. This compound facilitates the release of TGFα in vitro and in vivo and activates signaling pathways initiated by this growth factor. We conclude that compounds of this kind that stimulate neurogenesis may be useful to counteract the neurological effects of pathological aging.

## Graphical abstract

Diterpene ER272 rescues of adult hippocampal neurogenesis and cognitive performance in the aging brain.

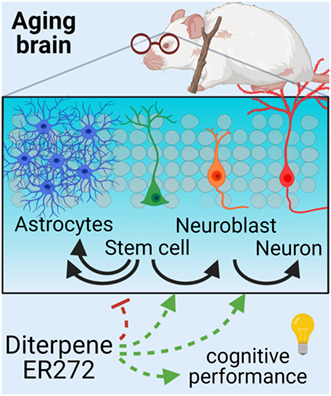

## 1 INTRODUCTION

Increasing age constitutes a risk factor for developing neurodegenerative disorders (Murman, 2015). In some individuals, age differentially affects distinct brain regions, mainly influencing memory (Gray & Barnes, 2015). Concretely, episodic memory is one of the most affected in the aging population (Nyberg et al., 2012). A form of plasticity that may help protect the brain during the continuous process of aging is the ability of some structures, including the hippocampal dentate gyrus (DG), to generate new neurons. Neurogenesis—the generation of neurons from neural stem cells—has been observed in the adult brain of many mammalian species, including humans, and throughout the individual's life (Aimone et al., 2014). The generated neurons influence cognitive ability by affecting tasks such as memory, learning, and pattern separation (Aimone et al., 2011; Deng et al., 2010). Although there still exists some controversy regarding the existence of neurogenesis in the adult human hippocampus and about its role in pathological aging (Moreno‐Jimenez et al., 2019; Sorrells et al., 2018), the development of new protocols (Flor‐Garcia et al., 2020) have led to additional studies that describe the existence of neurogenesis throughout adult life (Moreno‐Jimenez et al., 2019; Tobin et al., 2019) and indicate that this capacity is altered in patients with age‐associated neurodegenerative disorders (Marquez‐Valadez et al., 2022; Terreros‐Roncal et al., 2021). The generation of neurons from neural stem cells (NSC) requires an adequate physiological environment built up of cells, trophic factors, elements of the extracellular matrix, etc. that activate NSC and direct their fate toward neurons. This environment constitutes what is called a neurogenic niche. Like the rest of the organism neurogenic niches age, thus altering and reducing the ability of NSC to generate neurons (Diaz‐Moreno et al., 2018; Encinas et al., 2011). In general, several pieces of evidence show that hippocampal‐dependent cognitive abilities decline with age in mammals, including humans, with a concomitant reduction in adult hippocampal neurogenesis (Akers et al., 2014; Magavi et al., 2000; Yassa et al., 2011). A key point in the regulation of neurogenesis within neurogenic niches is the control of NSC activation. NSC may enter a prolonged quiescent state or enter an active state. NSC are exposed to a wide variety of environmental signals, either inhibitory or stimulatory. Depending on the result of the integration of these signals, the resting state (qNSC) is maintained or the transition to an activated state (aNSC) occurs. Once activated, DG NSC carries out a series of asymmetric divisions that produce neuron precursors until they eventually differentiate into astrocytes (Encinas et al., 2011); the ratio of NSC to neurons varies in the DG with age, and a trend toward astroglia differentiation can be observed in aged mice resulting in depletion of the NSC pool and reduced neurogenesis (Akers et al., 2014). Thus, the number of hippocampal NSC decreases with age, and at the same time, these cells undergo a transition to a senescent‐like state characterized by a complex morphology. The ability of these senescent cells to undergo activation is considerably reduced, remaining inactive for a longer time in the DG of older adults (Diaz‐Moreno et al., 2018; Martin‐Suarez et al., 2019). Recent reports show that gene expression of NSC changes in the aged DG to reduce NSC activation and ensure the maintenance of the stem cells population (Harris et al., 2021), and a pool of long‐term quiescent stem cells is found in the young brain that is preserved for long periods (Ibrayeva et al., 2021). Therefore, the aged niche negatively regulates qNSC activation to preserve the NSC pool reducing neurogenesis. Thus, the search for strategies that promote neurogenesis without depleting the NSC pool could be of use to develop treatments that alleviate the cognitive deterioration associated with aging.

In a previous work, we showed that a diterpene with 12‐deoxyphorbol structure (12‐desoxyphorbol 13‐isobutyrate: DPB or ER272), stimulated hippocampal neurogenesis in adult healthy mice (Dominguez‐Garcia et al., 2020), suggesting its ability to act as a pharmacological drug that promotes neurogenesis in mouse models with a compromised cognitive capacity. The mouse model with accelerated aging senescence‐accelerated mouse‐prone SAMP8 and its control senescence‐accelerated mouse resistant SAMR1 (Takeda, 2009) have long been used to study brain aging and its effects on cognitive ability and neurogenesis. In addition to multisystemic aging, this model presents precociously and progressively cognitive deterioration (Yagi et al., 1988) starting at 4 months and after 6 months it begins to develop typical characteristics of Alzheimer's disease (Butterfield & Poon, 2005; Dobarro et al., 2013; Pallas et al., 2008). Interestingly, in this model, an activation of neurogenesis can be observed at early stages, resulting in the subsequent depletion of the NSC reservoir caused by extracellular signals present in the niche (Diaz‐Moreno et al., 2013; Gang et al., 2011; Soriano‐Canton et al., 2015). We show here that chronic treatment of SAMP8 mice with ER272 for 2 months previous to the initiation of cognitive deterioration improves cognitive performance concomitantly increasing adult hippocampal neurogenesis. The analysis of the cellular and molecular mechanisms of action shows that the compound facilitates the activation of NSC and facilitates the release of the ligand of the epidermal growth factor receptor (EGFR), the transforming growth factor‐alpha (TGFα) in a mechanism dependent on classical protein kinase C alpha (PKCα).

## 2 METHODOLOGY

### 2.1 Reagents

Isolation and purification of the 12‐deoxyphorbol 13‐isobutyrate also referred to as DPB (Ezzanad et al., 2021) or ER272 (Dominguez‐Garcia et al., 2021; Geribaldi‐Doldan et al., 2015; CAS:25090‐74‐8) was performed in our laboratory as described in supplementary methods of Geribaldi‐Doldan et al. (2015). PKC inhibitors were purchased from Sigma‐Aldrich and Calbiochem (Millipore). SmartPool One target siRNA specific for each PKC was obtained from Horizon.

### 2.2 Animal subjects

Four‐month‐old SAMR1 and SAMP8 male mice were housed under controlled conditions of temperature (21–23°C) and light (LD 12:12) with free access to food (AO4 standard maintenance diet; SAFE) and water. Care and handling of animals were performed according to the Guidelines of the European Union Council (2010/63/EU), and the Spanish regulations (65/2012 and RD53/2013) for the use of laboratory animals. All studies involving animals are reported in accordance with the ARRIVE guidelines for reporting experiments involving animals (Kilkenny et al., 2010; McGrath et al., 2010).

### 2.3 Treatments and experimental groups

Four‐month SAMP8 mice were treated with either vehicle or ER272 administered intranasally (see below) during 8 weeks. SAMR1 mice were used as the control group as indicated in previous reports (Takeda, 2009). During the treatment period, mice received intraperitoneal injections of 5‐bromo‐2‐deoxyuridine (BrdU) every 2 days. We have used SAMR1 as a control of the model to understand the differences between the control and the SAMP8 model of neuropathological aging. Only SAMP8 mice have been treated to study whether ER272 prevented the cognitive and neurogenic alterations found in this mouse model. An experimental design of this kind has been used in previous works (Iwata et al., 2020; Shi et al., 2020).

### 2.4 Intranasal administration of ER272

ER272 was delivered intranasally while the animal was placed in a standing position with an extended neck as previously described (Marks et al., 2009). Eighteen microliters of each solution (1 μM ER272 in saline, or saline as vehicle) was delivered over both nasal cavities alternating 3 μL/each using a micropipette. Mouse was maintained in such position for 10 additional seconds to ensure all fluid was inhaled. In all experiments, mice were coded and treatment (vehicle or ER272) was assigned randomly to code numbers and applied. In addition, blind quantifications were performed to avoid subjective biases.

### 2.5 Motor activity and new object discrimination (NOD) task

Behavioral testing commenced 14 days before sacrifice. Motor activity was analyzed by measuring the distance traveled by each mouse during a 30‐min period before initiating the NOD test. One day after the object habituation training (a red cylinder and a yellow trapezoid), the NOD test begins. Then, integrated episodic memory for the paradigms “what”, “when”, and “where” was analyzed as described in previous reports (Ramos‐Rodriguez et al., 2013). In brief, next day animals were placed in the center of the box for 5 min with 2 objects (red cylinder and yellow trapezoid) for habituation purposes. The next day mice were placed in the block with four copies of a novel object (blue balls) arranged in a triangle‐shaped spatial configuration and allowed to explore them for 5 min. After a delay of 30 min, the mice received a second sample trial with four novel objects (red cones), arranged in a quadratic‐shaped spatial configuration, for 5 min. After a delay of 30 min, the mice received a test trial with two copies of the object from sample trial 2 (“recent” objects) placed at the same position, and two copies of the object from sample trial 1 (“familiar” objects) placed one of them at the same position (“old nondisplaced” object) and the another in a new position (“familiar displaced” object). Integrated episodic memory for “what” “where”, and “when” was analyzed as previously described (Dere et al., 2005; Infante‐Garcia et al., 2018; Segado‐Arenas et al., 2018): “What” was defined as the difference in time exploring familiar and recent objects, “where” was defined as the difference in time exploring displaced and nondisplaced objects and “when” was defined as the difference between time exploring familiar nondisplaced and recent nondisplaced objects.

### 2.6 Morris water maze (MWM)

Spatial memory and learning tasks were analyzed before sacrifice using the MWM test in control and treated mice the next day after the NOD test was concluded as previously described (Ramos‐Rodriguez et al., 2013). Briefly, the pool consisted of a round tank (95 cm in diameter) divided into four virtual quadrants. Geometrical shapes were located around the pool for orientation purposes. The scape platform was 2 cm below water surface in quadrant 2 and water temperature was 21 ± 1°C. The acquisition phase took place 4 consecutive days and animals received 4 trials/day with a 10‐min interval. Time limit was 60 s and animals were allowed to spend 10 s on the platform. If the animal did not reach the platform, it was placed on it for 10 s. The retention phase took place 24 h after concluding the acquisition phase. Platform was removed and mice were allowed to swim for 60 s. Time to reach the platform along the acquisition phase and the time spent in the quadrant where the platform used to be located were recorded and analyzed using SMART software (Panlab).

### 2.7 RNA isolation, reverse transcription, and real‐time quantitative PCR

For RT‐qPCR analysis, we follow the procedures previously used (Romero‐Grimaldi et al., 2011). In brief, RNA was isolated from intact hippocampi that were processed for RNA extraction using the TRIzol™ (Cat. 15596026; Invitrogen), separation method, following the manufacturer's instructions and resuspended in purified nuclease‐free water. RNA was quantified using BioTek's Synergy™ Mx fluorimeter (BioTek Instruments, Inc). cDNA was prepared from 500 ng RNA using iScript™ cDNA Synthesis Kit (Cat. 1708890; BioRad Laboratories Inc) on a Techne Genius thermal cycler (Techne Ltd.). The 15 μL RT‐qPCR reaction mix contained 7.5 μL 2× iTaq™ Universal SYBR® Green Supermix (Cat. 1725122; BioRad Laboratories Inc), 10 nmol of both the forward and the reverse primers, and 1 μL of the sample. The PCR thermal profile included 40 cycles of denaturation at 95°C for 10 s, an annealing temperature according to each set of primers for 15 s and extension at 72°C for 20 s, followed by a melting curve analysis. Each sample was analyzed in triplicate. The mRNA level of rRNA18S was used as an internal control. Relative quantification values of mRNA expression were calculated as $2^{-\Delta \Delta C_{t}}$ (Livak Method). Oligonucleotide primers used in this study were designed by BLAST and were obtained from Merck. Primer sequences (5′–3′) for detecting expression of mouse mRNA were the following: for TGFα, FW: CCAGATTCCCACACTCAGT, RW: GGAGGTCTGCATGCTCACA.

### 2.8 Cerebrospinal fluid extraction

Cerebrospinal fluid (CSF) collection was performed as described by Lim et al. (2018). Mice were anesthetized as described above and placed prone on the stereotaxic instrument. Muscles were moved to the side and dura mater over the cisterna magna was exposed. The capillary tube was placed and inserted into the *cisterna magna* through the *dura mater*, lateral to the *arteria dorsalis spinalis*. Finally, the CSF was collected.

### 2.9 Concentration of TGFα in CSF

TGFα was measured in the CSF using commercial ELISA kits, MBS2508394 (MyBioSourse, Inc), following the manufacturer's instructions. CSF was centrifugated for 20 min at 10 g and 4°C; then, the supernatant was collected. Blanks (diluent only) were included in each independent determination. Blanks were subtracted from measurements before comparisons were made.

### 2.10 Brain processing and immunohistochemistry

At the end of the treatment, brains were perfused with paraformaldehyde (PFA) and sliced using a cryotome into 30 μm sections. Immunohistochemistry was performed as previously described (Garcia‐Bernal et al., 2018; Geribaldi‐Doldan et al., 2015; Murillo‐Carretero et al., 2017). See antibodies in Table S1.

### 2.11 Quantification of neurogenesis in brain sections

Cells positive for BrdU, DCX, NeuN, GFAP, SOX2, and S100β in the DG were estimated as described (Rabaneda et al., 2008, 2016). After perfusion, mouse brains were coded and blind quantification was performed as previously described (Geribaldi‐Doldan et al., 2018). Positive cells were counted throughout the entire lateral or laterodorsal walls of the lateral ventricles in every fifth section containing the hippocampus; 14–16 sections were analyzed per brain under confocal microscopy (Zeiss LSM 900 Airyscan 2). Confocal imaging was taken every 1.50 μm in the Z‐plane using a 20× objective. Cell density was calculated for each section relative to the DG volume (mm^3^) and averaged for each animal as reported previously (Dominguez‐Garcia et al., 2021).

### 2.12 Morphological analysis

DCX^+^ cells 3D reconstructions from confocal stack images were obtained using Neurolucida 360 (MBF Bioscience) and the total dendritic length, total dendritic surface, total dendritic segments, number of terminal segments, and 3D Sholl analysis were analyzed with Neurolucida Explorer (MBF Bioscience) according to previous reports (Carrascal et al., 2009; Nunez‐Abades et al., 1994).

### 2.13 Western blot

Tissue was homogenized using a commercial lysis buffer (Cell Signaling) supplemented with a protease and phosphatase inhibitor cocktail (Sigma). The homogenates were sonicated and centrifuged at 4°C for 5 min at 16,000 g. Supernatants were collected and protein concentration was measured using the Pierce BCA Protein Assay Kit (Thermo Scientific). Equal amounts (30 μg) of total protein from each cellular extract were subjected to SDS‐PAGE and western blotting. Proteins were separated on gradient 4%–15% precast polyacrylamide gels (Mini‐PROTEAN TGX Stain‐Free Protein Gels, BioRad), followed by electrophoretic transfer to PVDF membranes (Schleicher & Schuell, Keene). Membranes were then soaked in blocking buffer (Invitrogen) for 30 min and incubated overnight at 4°C with primary antibodies (see antibodies in Table S1). Membranes were washed, and the signal was detected using commercial kits (Western Breeze, Invitrogen) containing either anti‐rabbit or anti‐mouse secondary antibodies conjugated to alkaline phosphatase, plus the corresponding chemiluminescent substrate following the manufacturer's instructions. Membranes were developed using Chemidoc Touch Imaging System 732BR1030 (BioRad).

### 2.14 HEK293T culture, cloning, and transfection

HEK293T obtained from ATCC were cultured and transfected as previously described (Geribaldi‐Doldan et al., 2018). After overnight incubation, cells were left for 30 min in serum‐free Fluorobrite DMEM (Thermo Fisher Scientific) and used either in time‐lapse experiments.

### 2.15 Cloning of human TGFα cDNA fused to eGFP and mCherry

Full‐length cDNA encoding the membrane‐bound isoform of human pro‐TGFα (TGFA, NCBI reference sequence: NM_003236.4) with mCherry cDNA inserted between nucleotides 126 and 127 of TGFA open reading frame was cloned into pEGFP‐N1 to add EGFP cDNA to the 3′ end. Construct was synthesized by GeneCust to generate the mCherry‐TGFα‐eGFP construct.

### 2.16 siRNA transfection

For the specific inhibition of classical PKCα and β cells were transfected with specific siRNA Smart Pool siRNA One target (Horizon). Cell transfection with TGFα construct and siRNA pool was performed at 18 h after seeding; for this, cells were changed to an antibiotic‐free medium and transfected using Lipofectamine 2000 (Invitrogen), following the manufacturer's instructions. Lipofectamine was removed 6 h later.

### 2.17 Time‐lapse experiments and fluorescence analysis of recombinant mCherry‐TGFα‐eGFP protein in the culture medium of HEK293T

HEK293T cells were plated in μ–dishes (35 mm high; Ibidi) and treated with ER272 and/or inhibitors, and images were taken as described in figure legends. Previous works have shown that HEK293T cells express all of the proteins required for the PKC‐dependent cleavage of TGFα mediated by ADAM17 (Dominguez‐Garcia et al., 2020).

### 2.18 Statistical analysis

The data and statistical analysis comply with the recommendations on experimental design and analysis in pharmacology (Curtis & Abernethy, 2015). Statistical analysis was performed using the computer program IBM SPSS Statistics 22. Unless otherwise indicated, the normal distribution of the data was first analyzed using a Shapiro‐Wilks test. Then, a Brown Forsythe test was performed to test the equality of variances. Afterward, when more than one treatment group was compared, statistical analyses were performed using one‐way ANOVA followed by a post hoc Tukey's test unless otherwise indicated. Two‐way ANOVA (group × day) was used in the acquisition phase of the MWM. Differences were considered significant at values of *p* < 0.05. In general, the sample size used in statistical analysis was *n* = 6–10 for in vivo experiments, *n* = 5–9 for in vitro experiments, and *n* = 9–12 in behavioral tests. Sample sizes were chosen based on previous works related to this one (Carrasco et al., 2014; Dominguez‐Garcia et al., 2021; Geribaldi‐Doldan et al., 2018; Murillo‐Carretero et al., 2017; Rabaneda et al., 2016).

## 3 RESULTS

**FIGURE 1:**
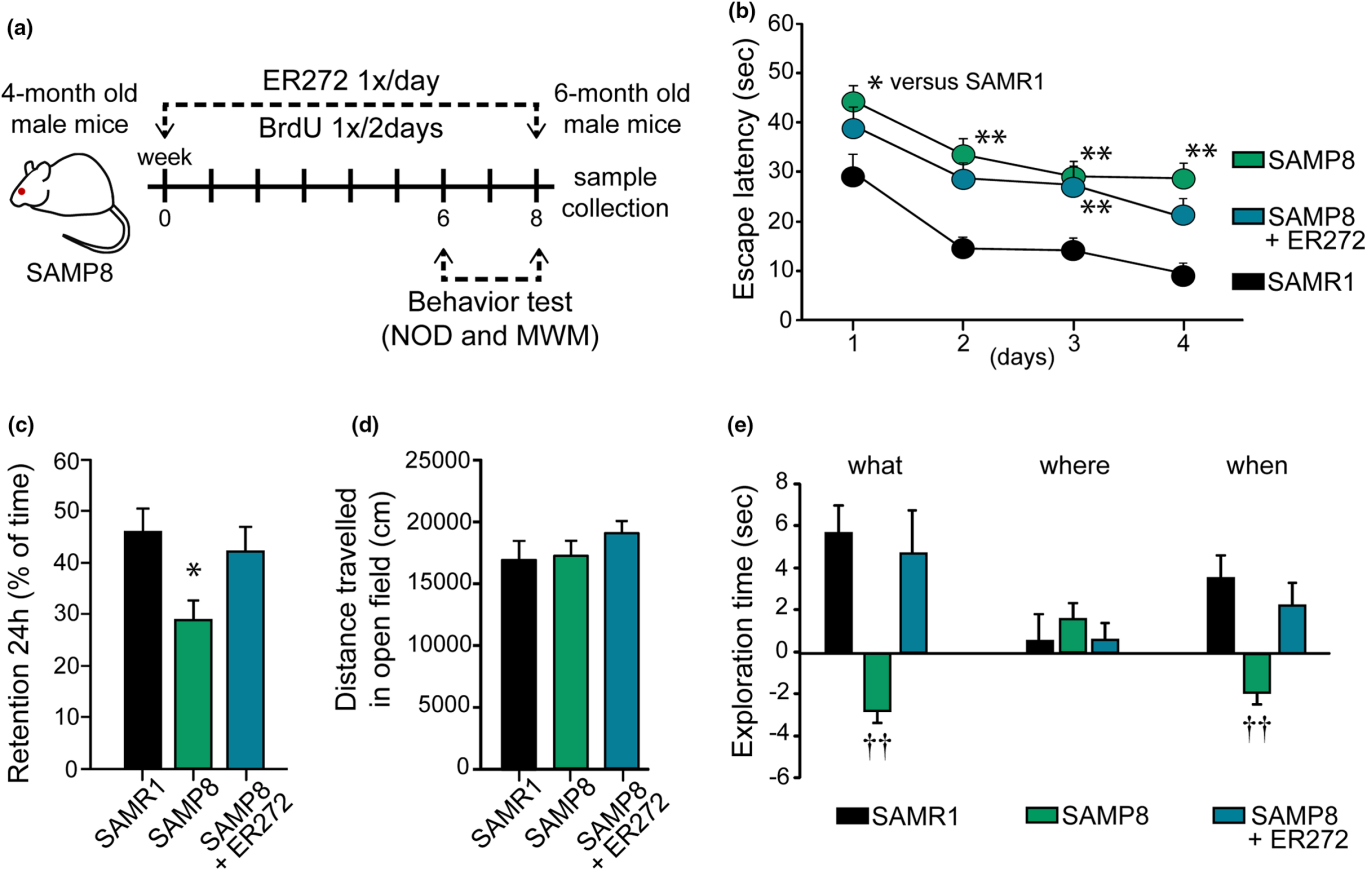
Treatment of SAMP8 mice with ER272 improves cognitive performance. (a) Experimental design. Four‐month‐old SAMP8 mice were treated with vehicle or ER272 (SAMP8+ER272) for 8 weeks until the age of 6 months. They were compared with SAMR1 mice of the same age treated with vehicle. Mice received BrdU during the 8‐week period every 2 days. Behavior tests took place during the last 2 weeks. (b) Scape latency (sec) in the MWM test. A compromise was observed in SAMP8 male mice when they were evaluated in the MWM and a slight improvement was observed for treated mice (SAMP8+ER272). Individual daily assessment revealed a better performance as training sessions progressed: day 1 [*F*_(2,116)_ = 4.64, *p = 0.031 vs. SAMR1], day 2 [*F*_(2,116)_ = 10.26, **p < 0.01 vs. SAMR1], day 3 [*F*_(2,113)_ = 3.67, **p < 0.001 vs. SAMR1], day 4 [*F*_(2,116)_ = 11.14, **p < 0.001 vs. SAMR1]. (c) Retention test in the MWM. In the 24 h retention phase, SAMP8 male mice spent shorter times in the quadrant where the platform used to be located (quadrant 2) [*F*_(2,25)_ = 4.16, *p = 0.028 vs. SAMR1]. The treatment reverted this situation. (d) Distance travel in the open field test. No differences were observed among groups when distances traveled in the open field were analyzed [*F*_(3,38)_ = 1.83, *p* = 0.158]. (e) NOD test. Episodic memory was severely affected in male SAMP8 mice when “what” and “when” paradigms were assessed, while ER272 treatment counterbalanced this situation. “What” [*F*_(2,84)_ = 9.55, ††*p* < 0.001 vs. rest of the groups], “where” [*F*_(2,84)_ = 0.309, *p* = 0.735], “when” [*F*_(2,89)_ = 8.69, ††*p* < 0.001 vs. rest of the groups]. Data are the mean ± SEM of 10 animals, *n* = 10. Differences detected by one‐way ANOVA followed by the Tukey *b* test

In order to test the effects of ER272 on adult hippocampal neurogenesis and cognitive performance in the SAMP8 mouse model, we used the experimental design represented in the diagram included in Figure 1a. Four‐month‐old SAMP8 mice were treated with either vehicle or ER272 during 8 weeks. SAMR1 mice were used as the control group as indicated in previous reports (Takeda, 2009). During the treatment period, mice received intraperitoneal injections of 5‐bromo‐2‐deoxyuridine (BrdU) every other day. On week 6, behavioral tests were performed (Morris water Maze, MWM; new object discrimination, NOD; and open field), being sacrificed at the end of the 8th week.

### 3.1 Long‐term intranasal administration of ER272 improves cognitive performance in SAMP8 mice

As described previously, spatial memory was compromised in male SAMP8 mice as reflected in the evaluation on the MWM and a slight improvement was observed in SAMP8 mice treated with ER272. No significant group × day effect was detected [*F*_(__2,462__)_ = 0.358, *p* = 0.905]; however, individual daily assessment revealed a better performance of treated mice as training sessions progressed (Figure 1b). In the retention phase of the MWM, we observed that SAMP8 mice spent significantly shorter times in the quadrant where the platform used to be located when compared with SAMR1 mice. Interestingly, in the retention phase, SAMP8 mice treated with ER272 did not show differences in comparison with the SAMR1 control group (Figure 1c). We specifically addressed the effect of ER272 treatment by comparing SAMP8 and SAMP8‐ER272 animals in behavioral studies. As described when all three groups were compared, no significant day × group effect was observed when SAMP8 and SAMP8‐ER272 groups were compared in the acquisition phase of the MWM [*F*_(3,306)_ = 0.393, *p* = 0.758]. Differences did not reach statistical significance when individual days were analyzed (day 1 [*F*_(3,77)_ = 1.561, *p* = 0.215], day 2 [*F*_(3,77)_ = 0.907, *p* = 0.344], day 3 [*F*_(3,74)_ = 0.013, *p* = 0.908], day 4 [*F*_(3,78)_ = 2.620, *p* = 0.110] (Figure 1b)). Nevertheless, the slightly better performances observed in SAMP8‐ER272 mice along the acquisition phase were enough to significantly improve the performance in the retention phase, and SAMP8‐ER272 mice spent significantly longer times in the quadrant where the platform used to be located, when compared with SAMP8 animals (*p = 0.041) (Figure 1c). As expected, episodic memory was severely affected in male SAMP8 mice when “what” and “when” paradigms were assessed (*p* < 0.001), while ER272 treatment counterbalanced this situation. On the contrary, no differences were observed in the “where” paradigm (Figure 1e). When we compared SAMP8 and SAMP8‐ER272 performances in the NOD test, we observed an overall improvement in treated animals (what **p = 0.001, when *p* = 0.411 and where **p = 0.002) supporting the beneficial effect of ER272 in SAMP8 animals. As control, no differences were observed in the distance traveled in the open field (Figure 1d).

### 3.2 Long‐term intranasal administration of ER272 promotes neurogenesis in the DG of SAMP8 mice

**FIGURE 2:**
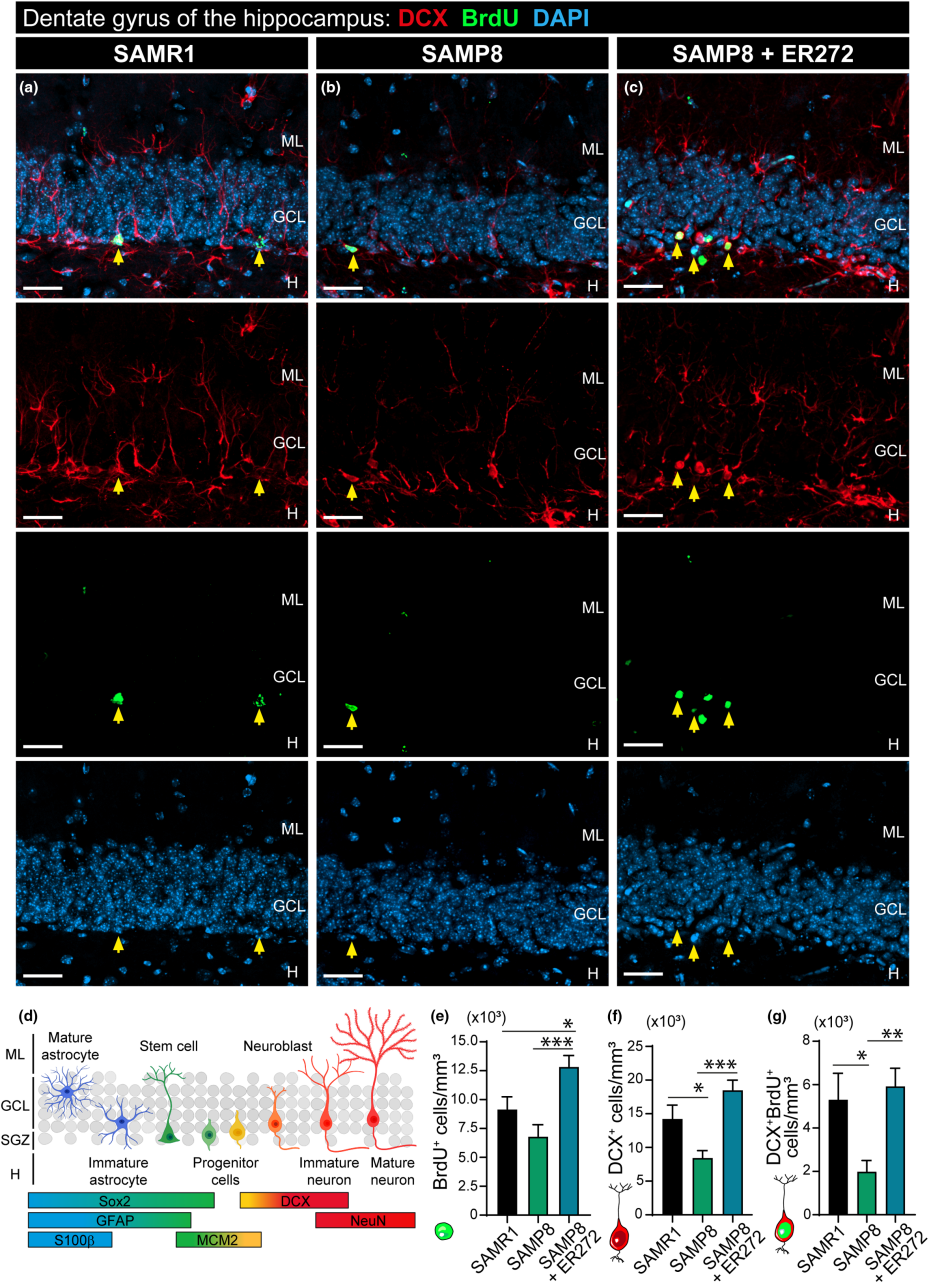
Effect of long‐term intranasal administration of ER272 to SAMP8 mice on DCX^+^ neuroblasts. (a–c) Representative confocal microscopy images of the DG of the hippocampus of 6‐month‐old SAMR1 and SAMP8 male mice treated with vehicle (a, b, respectively) or SAMP8 mice treated with ER272 (SAMP8+ER272) (c) during 8 weeks as indicated in Figure 1a. Slices were processed for the immunohistochemical detection of the proliferation marker BrdU (lower medium panel; green) and DCX (upper medium panel; red). DAPI staining is shown in blue (lower panel). Merged channels are shown in the upper panel. Yellow arrows indicate DCX^+^BrdU^+^DAPI^+^ cells. (d) Schematic drawing showing the hierarchical model of adult hippocampal neurogenesis indicating the different markers along the cell lineage within the dentate gyrus and showing molecular layer (ML), granular cell (GCL), subgranular cell zone (SGZ), and hilus (H). (e) Graph shows the total number of BrdU^+^ nuclei in the DG of the hippocampus per mm^3^ [*F*_(2,32)_ = 10.58, *p = 0.045 SAMR1 vs. SAMP8+ER272] [*F*_(2,32)_ = 10.58, ***p < 0.001 P8 vs. P8+ER272]. (f) Graph shows the total number of DCX^+^ in the DG of the hippocampus per mm^3^ [*F*_(2,29)_ = 10.88, *p = 0.036 SAMR1vs. P8] [*F*_(2,29)_ = 10.88, ***p < 0.001 SAMP8 vs. SAMP8+ER272]. (g) Graph shows the total number of BrdU^+^DCX^+^ cells in the DG of the hippocampus per mm^3^ [*F*_(2,31)_ = 6.898, *p = 0.034 SAMR1 vs. P8] [*F*_(2,31)_ = 6.898, **p < 0.003 P8 vs. SAMP8+ER272]. Data are the mean ± SEM of six animals, *n* = 6. Differences detected by one‐way ANOVA followed by the Tukey *b* test. Scale bar represents 25 μm.

**FIGURE 3:**
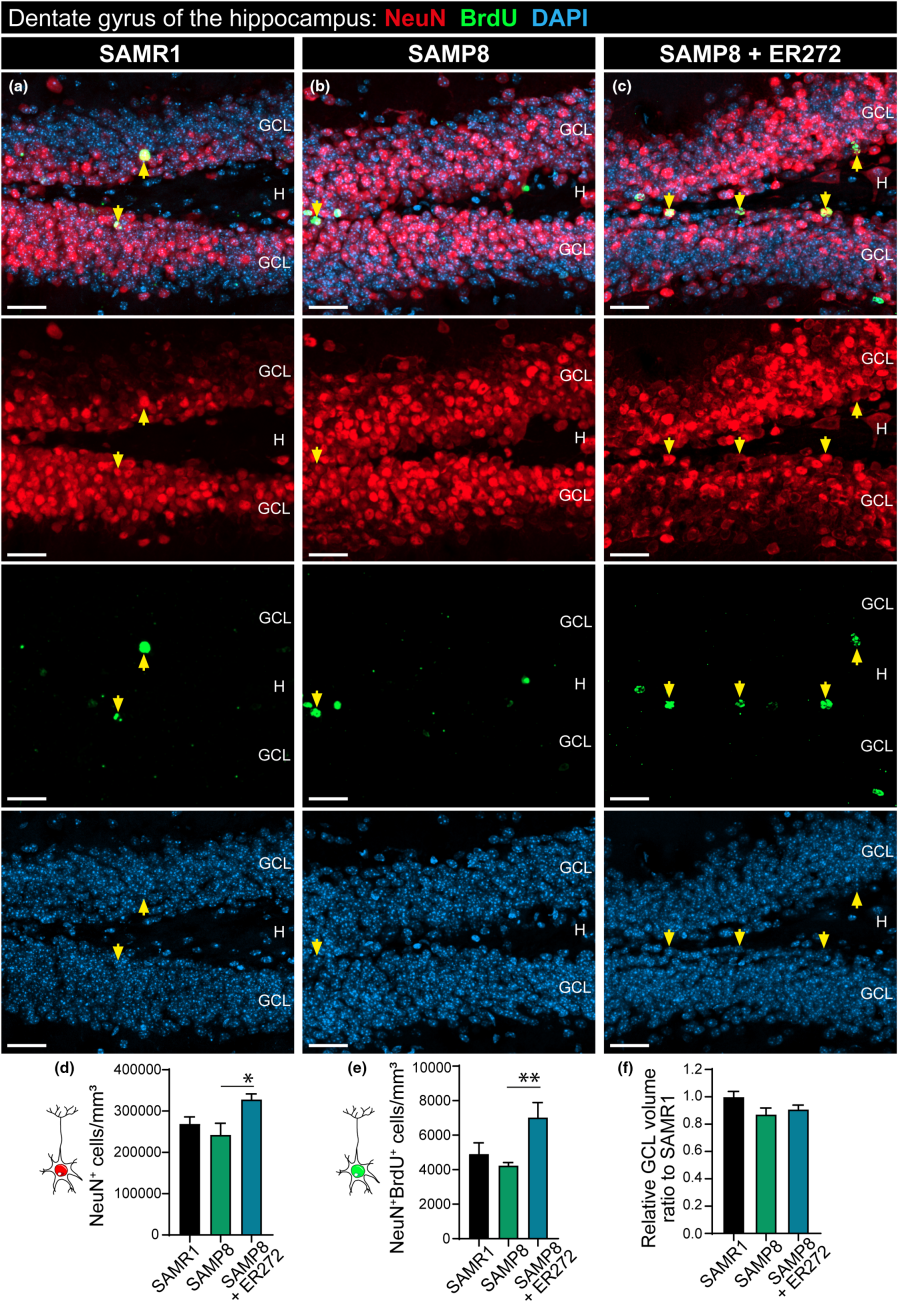
Effect of long‐term intranasal administration of ER272 to SAMP8 mice on NeuN^+^ cells. (a–c) Representative confocal microscopy images of the DG of the hippocampus of six‐month‐old SAMR1 and SAMP8 male mice treated with vehicle (a, b, respectively) or SAMP8 mice treated with ER272 (c) during 8 weeks as indicated in Figure 1a. Slices were processed for the immunohistochemical detection of the proliferation marker BrdU (lower medium panel; green) and NeuN (upper medium panel; red). DAPI staining is shown in blue (lower panel). Merged channels are shown in the upper panel. Yellow arrows indicate NeuN^+^BrdU^+^DAPI^+^ cells. (d) Graph shows the number of NeuN^+^ nuclei in the DG of the hippocampus per mm^3^ [*F*_(2,14)_ = 3.934, *p = 0.038 P8 vs. SAMP8+ER272]. (e) Graph shows the number of NeuN^+^BrdU^+^ nuclei in the DG of the hippocampus per mm^3^ [*F*_(2,8)_ = 11.04, **p = 0.004 P8 vs. SAMP8+ER272]. (f) The volume of the granular cell layer (CGL) in SAMP8 and SAMP+ER272‐treated mice compared with SAMR1 mice. Data are the mean ± SEM of six animals, *n* = 6. Differences detected by one‐way ANOVA followed by the Tukey *b* test. Scale bar represents 25 μm.

The effect of ER272 on the NSC progeny within the DG was analyzed using the markers indicated in Figure 2d. The analysis of BrdU^+^ cells did not show a reduction in the total number of cells when SAMP8 mice were compared with SAMR1 (Figure 2a,b,e and see Figure S1 for orthogonal projections). However, the DG of SAMP8‐treated mice showed a 2‐fold increase in the number of cells that had incorporated BrdU over the course of the treatment compared with SAMP8 mice (Figure 2a–c,e). The analysis of total DCX^+^ cells in all groups revealed that at 6 months of age, SAMP8 mice showed half the number of DCX^+^ cells (Figure 2a,b,f and see Figure S1 for orthogonal projections) and DCX^+^NeuN^+^ (Figure S2A–D) cells compared with SAMR1. Interestingly, the treatment of SAMP8 mice with ER272 increased the number of DCX^+^ (Figure 2b,c,f and see Figure S1 for orthogonal projections) and DCX^+^NeuN^+^ cells (Figure S2A–D) by 2‐fold compared with SAMP8 compensating this reduction. Identically, the number of DCX^+^ cells that had incorporated BrdU over the course of 2 months was dramatically reduced in SAMP8 mice compared with control (Figure 2a,b,g and see Figure S1 for orthogonal projections), whereas this number increased by 2‐fold in mice treated with ER272 (Figure 2b,c,g and see Figure S1 for orthogonal projections). Interestingly, the number of NeuN^+^ cells was not statistically different between the control and SAMP8 groups at 6 months of age; however, a significant 17% increase in the number of NeuN^+^ cells was observed in mice treated with ER272 (Figure 3a–d). In addition, a 40% increase in the number of NeuN^+^ cells that had incorporated BrdU was observed (Figure 3a–c,e). The increase in NeuN^+^ cells did not alter the volume of the granular cell layer (GCL; Figure 3f).

### 3.3 Morphology of DCX ^+^ cells is altered in SAMP8 mice and restored by ER272 treatment

**FIGURE 4:**
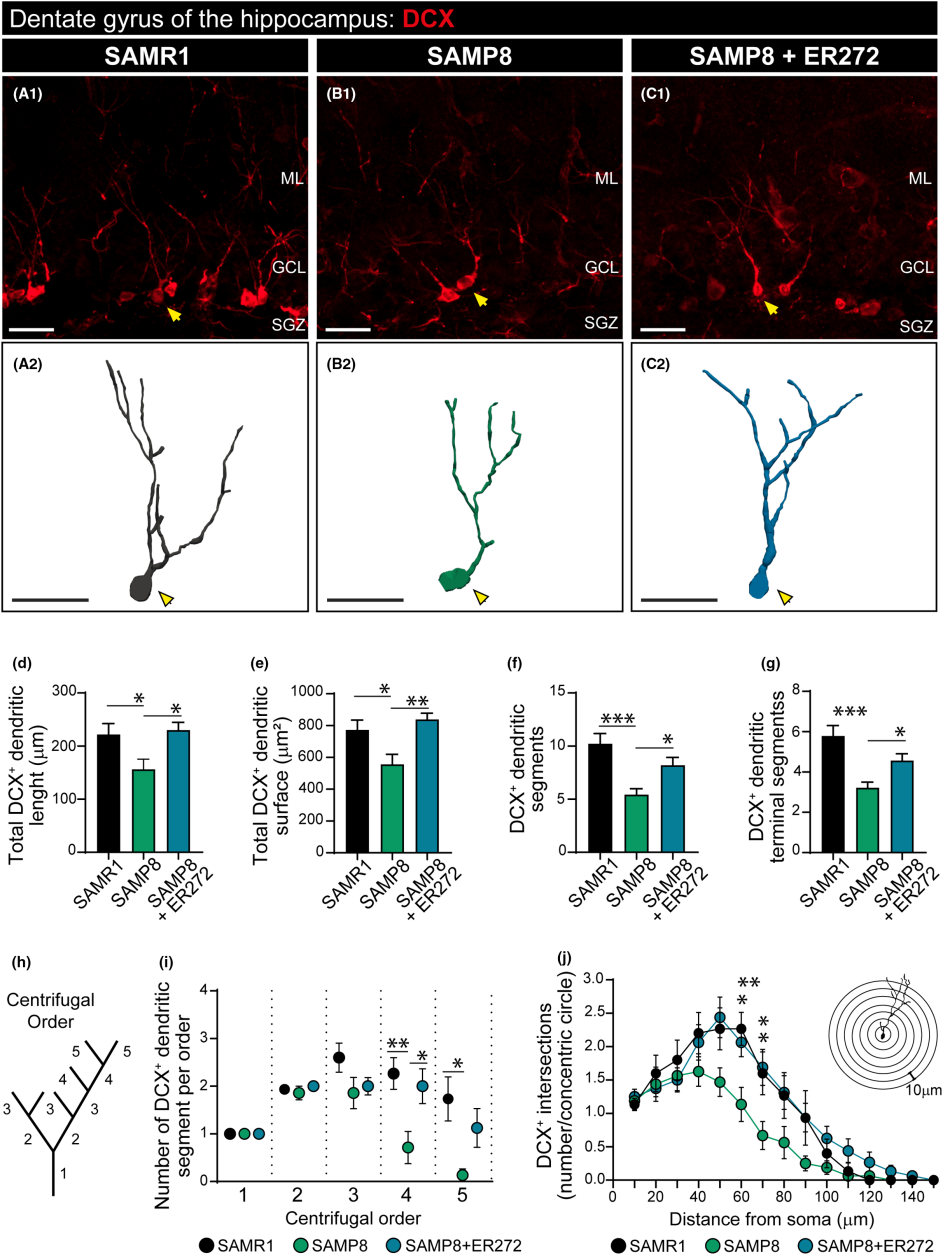
Altered morphology of DCX^+^ cells in SAMP8 is restored by ER272 treatment. (a–c) Representative confocal microscopy images of the DG of the hippocampus of six‐month‐old SAMR1 and SAMP8 male mice treated with vehicle (a1, b1, respectively) or SAMP8 mice treated with ER272 (SAMP8+ER272) (c1) during 8 weeks. Examples of the reconstructed DCX^+^ cells used in the analysis of SAMR1 and SAMP8 treated with vehicle (a2, c2, respectively) and SAMP8 treated with ER272 (c2). Yellow arrows indicate DCX^+^ cells used as 3D representative reconstruction. (d–g) Total dendritic length [*F*_(2,43)_ = 4.94, *p = 0.040 SAMR1 vs. SAMP8] [*F*_(2,43)_ = 4.94, *p = 0.016 SAMP8 vs. SAMP8+ER272] (d), total dendritic surface [*F*_(2,43)_ = 7.157, *p = 0.024 SAMR1 vs. SAMP8] [*F*_(2,43)_ = 7.157, **p = 0.002 SAMP8 vs. SAMP8+ER272] (e), total dendritic segments [*F*_(2,40)_ = 9.51, ***p < 0.001 SAMR1 vs. SAMP8] [*F*_(2,40)_ = 9.51, *p = 0.038 SAMP8 vs. SAMP8+ER272] (f) and the number of terminal segments [*F*_(2,41)_ = 10.5, ***p < 0.001 SAMR1 vs. SAMP8] [*F*_(2,41)_ = 10.5, *p = 0.045 SAMP8 vs. SAMP8+ER272] (g) were measured using Neurolucida Explorer. (h) Scheme representing the centrifugal order of different dendritic segments. (i) Number of DCX^+^ segments in the different centrifugal orders. Quaternary order [*F*_(2,42)_ = 5.50, **p < 0.009 SAMR1 vs. SAMP8] [*F*_(2,42)_ = 5.50, *p = 0.032 SAMP8 vs. SAMP8+ER272]. Quinary order [*F*_(2,43)_ = 4.76, *p < 0.011 SAMR1 vs. SAMP8]. (j) Sholl analysis showing the number of intersections of the DCX^+^ dendritic segments at different distances from soma of cells. 60 μm from soma [*F*_(2,43)_ = 6.292, **p < 0.005 SAMR1 vs. SAMP8] [*F*_(2,43)_ = 6.292, *p < 0.022 SAMP8 vs. SAMP8+ER]. 70 μm from soma [*F*_(2,42)_ = 4.917, *p < 0.026 SAMR1 vs. SAMP8] [*F*_(2,42)_ = 4.917, *p < 0.025 SAMP8 vs. SAMP8+ER]. A total of 15 cells were analyzed per group. Data are the mean ± SEM of 15 cells per group, *n* = 15. Differences detected by one‐way ANOVA followed by the Tukey *b* test. Scale bar represents 25 μm.

The study of the morphological properties of DCX^+^ cells revealed that the total dendritic length, the total dendritic surface, the total dendritic segments, and the number of terminal segments were reduced in SAMP8 mice compared with control (Figure 4a–c,d–g). Also, the number of dendritic segments on the cuaternary and quinary centrifugal order was reduced in SAMP8 mice compared with SAMR1 (Figure 4a–c,h,i). It was very interesting to note that ER272 treatment reverted all these age‐induced morphological alterations (Figure 4a–i). In addition, Sholl analysis shows a lower number of intersections at distances from soma of 60–70 μm in SAMP8 mice compared with control (Figure 4j), an effect that was reverted by the ER272 treatment.

### 3.4 Effect of ER272 treatment on niche astrocytes

**FIGURE 5:**
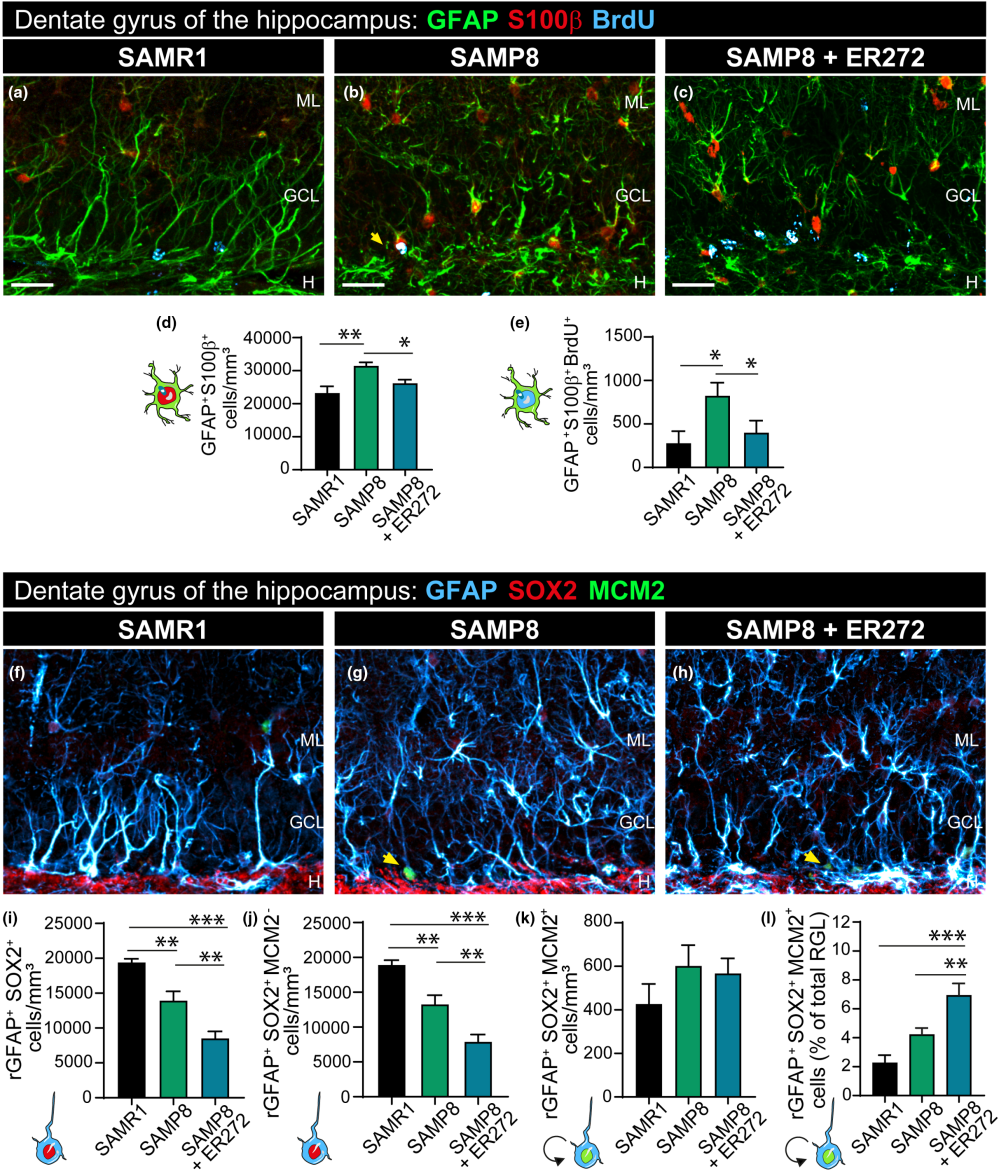
Intranasal administration of ER272 decreases the number of newly generated astrocytes in the dentate gyrus of SAMP8 mice and promotes the radial glial‐like cell activation in the dentate gyrus of SAMP8 mice. (a–c) Representative confocal microscopy images of the DG of the hippocampus of six‐month‐old SAMR1 and SAMP8 male mice treated with vehicle (a, b, respectively) or SAMP8 mice treated with ER272 (SAMP8+ER272) (c) during 8 weeks as indicated in Figure 1a. Slices were processed for the immunohistochemical detection of the proliferation marker BrdU (cyan), the Glial Fibrillary Acidic Protein (GFAP) (green), and the marker for astrocytes S100β (red). Yellow arrows indicate GFAP^+^S100β^+^BrdU^+^ cells. (d) Graph shows the number of GFAP^+^S100β^+^ cells in the DG per mm^3^ [*F*_(2,23)_ = 8.55, **p = 0.001 SAMR1 vs. SAMP8] [*F*_(2,23)_ = 8.55, *p < 0.05 SAMP8 vs. SAMP8+ER272]. (e) Graph shows the number of GFAP^+^S100β^+^BrdU^+^ cells in the DG of the hippocampus per mm^3^ [*F*_(2,13)_ = 5.60, *p < 0.0204 SAMR1 vs. SAMP8] [*F*_(2,13)_ = 5.60, *p < 0.0495 SAMP8 vs. SAMP8+ER272]. (f–h) Representative confocal microscopy images of the DG of the hippocampus of six‐month‐old SAMR1 and SAMP8 male mice treated with vehicle (f, g, respectively) or SAMP8 mice treated with ER272 (h) during 8 weeks as indicated in Figure 1a. Slices were processed for the immunohistochemical detection of the Glial Fibrillary Acidic Protein GFAP (cyan), SOX2 (red), and the cell‐cycle marker MCM2 (green). Yellow arrows indicate rGFAP^+^MCM2^+^SOX2^+^ cells. (i) Graph shows the total number of rGFAP^+^SOX2^+^ with a radial glial‐like cell (r) morphology in the DG per mm^3^ [*F*_(2,15)_ = 27.9, **p < 0.005 SAMR1 vs. SAMP8] [*F*_(2,15)_ = 27.9, ***p < 0.001 SAMR1 vs. SAMP8+ER272] [F_(2,15)_ = 27.9, **p = 0.005 SAMP8 vs. SAMP8+ER272]. (j) Graph shows the number of rGFAP^+^SOX2^+^MCM2^−^ with a radial glial‐like cell (r) morphology in the DG per mm^3^ [*F*_(2,15)_ = 30.5, **p < 0.003 SAMR1 vs. SAMP8] [*F*_(2,15)_ = 30.5, ***p < 0.001 SAMR1 vs. SAMP8+ER272] [*F*_(2,15)_ = 30.5, **p = 0.005 SAMP8 vs. SAMP8+ER272]. (k) Graph shows the number of rGFAP^+^SOX2^+^MCM2^+^ radial glial‐like cells in the DG of the hippocampus per mm^3^. (l) Graph shows the percentage of rGFAP^+^SOX2^+^MCM2^+^ radial glial‐like cells per rGFAP^+^SOX2^+^ in the DG of the hippocampus [*F*_(2,15)_ = 14.3, ***p < 0.001 SAMR1 vs. SAMP8+ER272] [*F*_(2,15)_ = 14.3, *p = 0.019 SAMP8 vs. SAMP8+ER272]. Data are the mean ± SEM of six animals, *n* = 6. Differences detected by one‐way ANOVA followed by the Tukey *b* test. Scale bar represents 25 μm.

We next investigated whether astrogliogenesis was altered in these senescent mice and whether ER272 exerted any effect on the generation of new astrocytes. Results show a larger number of astrocytes expressing the glial marker protein glial fibrillary acidic protein (GFAP) and the mature astrocyte marker S100β   (GFAP^+^S100β^+^ cells) in SAMP8 mice compared with SAMR1 (Figure 5a–d and see Figure S3 for orthogonal projections) and S100β^+^SOX2^+^ (Figure S4A–D). In addition, a two‐fold increase in the number of astrocytes generated during the 2‐month period (GFAP^+^S100β^+^BrdU^+^cells and SOX2^+^S100β^+^BrdU^+^) was observed (Figure 5a–e and see Figure S3 for orthogonal projections; Figure S4A–E). Interestingly, the treatment with ER272 prevented the increase in the number of astrocytes (total and newly generated; Figure 5a–e and see Figure S3 for orthogonal projections; Figure S4A–E).

### 3.5 Effect of ER272 treatment on neural stem cells (radial glial‐like cells, rNSC)

We next investigated whether the treatment affected the NSC and its activation. Particularly we analyzed the number of cells that expressed (GFAP) and the transcription factor SOX2 that showed a radial glial‐like morphology (rGFAP^+^SOX2^+^). We could observe that the total number of rGFAP^+^SOX2^+^ cells decreased in SAMP8 mice compared with SAMR1 mice. Also, the treatment of SAMP8 mice with ER272 reduced this number (Figure 5f–i and see Figure S5 for orthogonal projections). Identically, the number of quiescent NSC (rGFAP^+^SOX2^+^MCM2^−^) was reduced in a similar manner (Figure 5f–h,j and see Figure S5 for orthogonal projections). To analyze the proportion of activated NSC undergoing the cell cycle, we studied the number of rGFAP^+^SOX2^+^ that expressed the mitosis marker MCM2 (rGFAP^+^SOX2^+^MCM2^+^). We did not observe a higher number of rGFAP^+^SOX2^+^MCM2^+^ cells in SAMP8 compared with control on the 6th month of age neither the proportion of rGFAP^+^SOX2^+^MCM2^+^ cells was different in SAMP8 mice compared with SAMR1 (Figure 5f–h,k; see Figure S5 for orthogonal projections). Nevertheless, the treatment with ER272 did not change the number of rGFAP^+^SOX2^+^MCM2^+^ cells in SAMP8 treated with ER272 in comparison with SAMP8, but it significantly increased the proportion of rGFAP^+^SOX2^+^MCM2^+^ of the total rGFAP^+^SOX2^+^ (Figure 5f–h,l; see Figure S5 for orthogonal projections).

### 3.6 ER272 promotes TGFα release in a reaction dependent on classical PKCα

**FIGURE 6:**
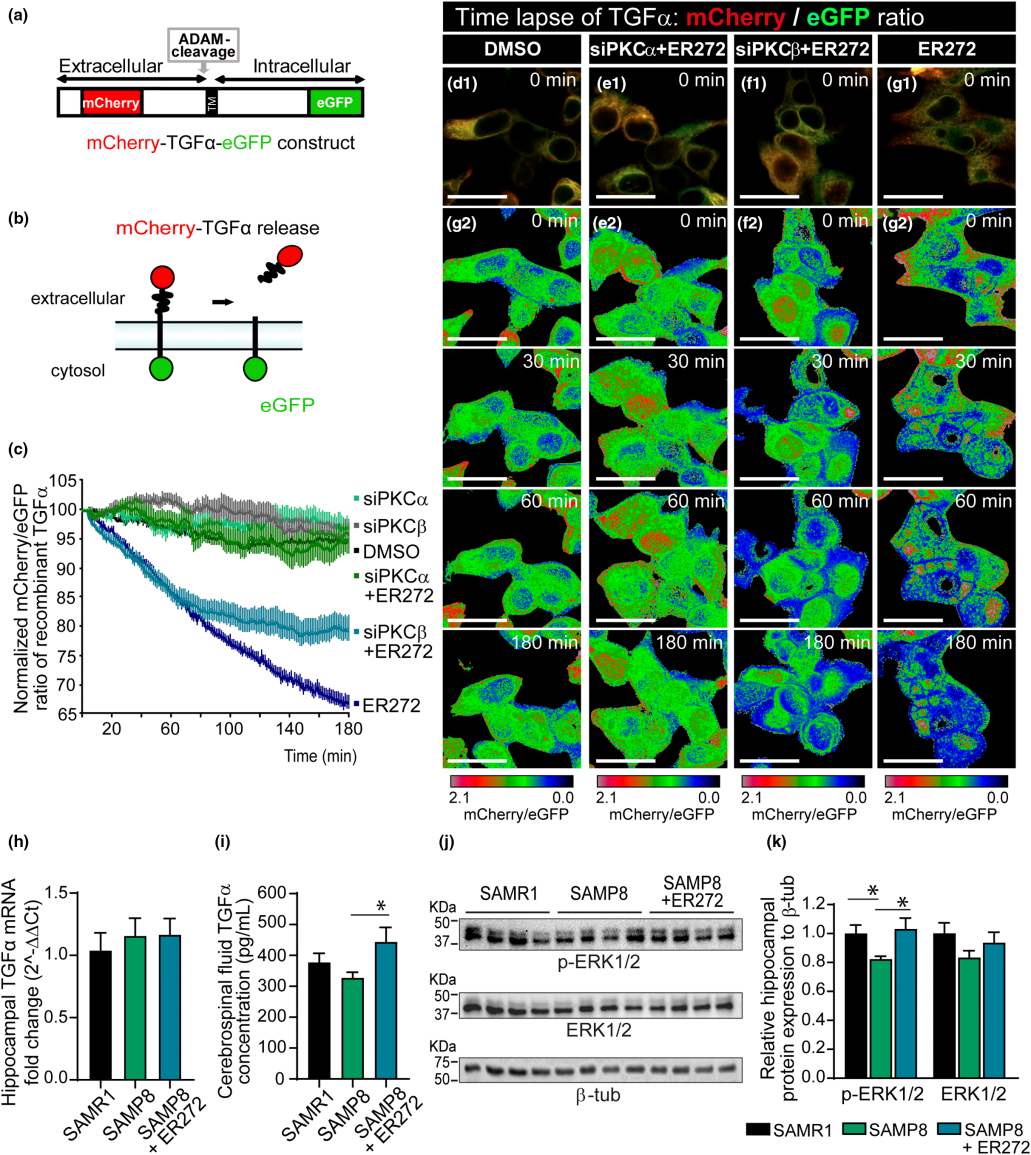
Molecular mechanisms underlying the effects of ER272: role of TGFα. (a) Scheme of mCherry‐TGFα‐eGFP construct (b) Mechanisms of TGFα‐bound fluorescence release. (c) Quantitative analysis of the microscopic images obtained from the time‐lapse assays of HEK293T cells expressing mCherry‐TGFα‐eGFP and stimulated with ER272, PKCα siRNA, and/or PKCβ siRNA. mCherry/eGFP ratios were normalized to the average mCherry/eGFP ratio measured before stimulation. The mean normalized mCherry/eGFP ratios and SEM are shown, *n* = 40. See also Movie S1. (d–g) mCherry/eGFP ratio images at the indicated time points are shown in the intensity‐modulated display mode. The color range goes from red to blue to represent mCherry/eGFP ratios. The upper and lower limits of the ratio range are shown on the right. Scale bar represents 25 μm. (h) Graph representing the relative expression of TGFα. $2^{\Delta \Delta C_{T}}$ values are represented relative to SAMR1. (i) Concentration of TGFα in the cerebrospinal fluid (CSF) measured by ELISA [*F*_(2,9)_ = 4.09, *p = 0.045 SAMP8 vs. SAMP8+ER272]. (j) Images of chemiluminescence signal of immunoblot detection of pERK1/2, total ERK1/2, and β‐tubulin (loading control) in the hippocampus of SAMR1, SAMP8 treated with vehicle and SAMP8 treated with ER272 (SAMP8+ER272). (k) Integrated optical density quantification of images obtained from immunoblots (see Figure S6): hippocampal protein expression of pERK1/2 [*F*_(2,17)_ = 6.59, *p = 0.029 SAMR1 vs. SAMP8] [*F*_(2,17)_ = 6.59, *p = 0.010 SAMP8 vs. SAMP8+ER272] and total ERK1/2 according to β‐tubulin. Data in j are the mean ± SEM of eight animals, *n* = 8. Differences detected by one‐way ANOVA followed by the Tukey *b* test

Previous reports had indicated that activation of protein kinase C facilitated the ADAM17‐mediated release of TGFα in vitro. To understand the mechanism of action of ER272, we studied its capacity to facilitate the release of the growth factor TGFα using time‐lapse imaging. As shown in Figure 6, pro‐TGFα was cloned in a mammalian expression vector flanked by red fluorescent protein in the N‐terminal portion and eGFP in the C‐terminal (Figure 6a,b). We transfected cultures of HEK293T cells with this construct, and using time‐lapse imaging, we quantified the release of the TGFα ligand from the pro‐ligand in the presence or absence of ER272 and in cultures co‐transfected with siRNA to block the expression of different classical PKC isozymes. The release of TGFα results in the loss of red fluorescence and therefore, in a reduction in the red/green fluorescence ratio (Figure 2b). As shown in Figure 6c–g, the addition of ER272 to cultures expressing the double fluorescent protein labeled construct, dramatically reduced the ratio of red/green over the course of 3 h (Figure 6c,g and Movie S1), whereas no changes were observed when the vehicle was added to the culture (Figure 6c,d and Movie S1). The addition of ER272 to cells in which PKCα expression had been abolished did not show any change in the fluorescent ratio (Figure 6c,e and Movie S1) and the addition of ER272 to cells in which PKCβ expression had been inhibited showed an intermediate effect on the red/green fluorescence ratio (Figure 6c,f and Movie S1).

### 3.7 Effect of ER272 on intracerebral concentration of TGFα and TGFα‐initiated signaling events

The hippocampal expression of TGFα was then investigated finding that no alterations on TGFα expression were found in any of the experimental groups (Figure 6h). Thus, we next investigated the effect of ER272 on the release of TGFα in vivo. To do that, we extracted cerebrospinal fluid (CSF) from the *cisterna magna* of SAMR1, SAMP8, and SAMP8‐treated mice before sacrifice and analyzed the concentration of TGFα in the CSF by ELISA immunodetection (Figure 6i). We observed that the concentration of TGFα in the CSF of mice treated with ER272 was significantly higher than in control and SAMP8 groups (Figure 6h). Also, the analysis by western blot of the activation of the Map kinase pathway, by looking at ERK1/2 phosphorylation, revealed that the phosphorylation of ERK1/2 is reduced in SAMP8 mice compared with SAMR1 mice and the treatment with ER272 reverts this effect (Figure 6j,K and Figure S6).

## 4 DISCUSSION

Increasing age is linked to cognitive decline and it is an independent risk factor for the development of neurodegenerative disorders (Murman, 2015). Specific types of memory have been found affected in aged individuals (Gray & Barnes, 2015), episodic memory being most affected in the aging population (Nyberg et al., 2012). The search for therapeutic strategies that prevent cognitive decline is nowadays a matter of crucial significance. In this context, strategies targeting the positive regulation of adult hippocampal neurogenesis may result in the development of new treatments to delay cognitive decline in the elderly. We had previously reported the isolation of molecules that promote neurogenesis in the adult brain and suggested its use as pharmacological tools to treat cognitive deficiencies related to a reduction in neurogenesis. A molecule of this type is the diterpene with 12‐deoxyphorbol structure ER272 (Geribaldi‐Doldan et al., 2015; Murillo‐Carretero et al., 2017). This molecule facilitates the secretion of trophic factors in vitro that act on receptor tyrosine kinases of the ErbB1‐4 family (Dominguez‐Garcia et al., 2020).

To analyze the effect of ER272 on a murine model of pathological aging we have used the senescence model SAMP8 using as a control for the SAMR1 strain (Takeda, 2009). SAMP8 recapitulates the transition from healthy aging to Alzheimer's disease. These mice spontaneously develop memory and learning deficiencies (Takeda, 2009) exhibiting Alzheimer's disease‐like features: oxidative stress, increased amyloid precursor protein (APP) and its mRNA, elevated amyloid β (Aβ) levels, phosphorylation of tau, and astrogliosis among others starting at 6 months of age (Butterfield & Poon, 2005; Diaz‐Moreno et al., 2013; Morley et al., 2000; Pallas et al., 2008). Studies on the neurogenesis in SAMP8 mice reveal that they display a proliferative response that precedes the Alzheimer's disease phenotype (Diaz‐Moreno et al., 2013) and an accelerated depletion of the hippocampal NSC pool compared with SAMR1 that coincides in space and time with an increase in astroglial differentiation and a reduction in neurogenesis (Diaz‐Moreno et al., 2018).

Using this model, we show that as previously described, SAMP8 mice show cognitive impairment (Takeda, 2009), particularly, we show that spatial memory is compromised showing difficulties to perform in the MWM test. Similarly, episodic memory in these mice is also impaired as it can be inferred from their performance in the NOD test. These results agree with previous results, which show similar memory impairments in this model (Dobarro et al., 2013). Interestingly, treatment of SAMP8 mice with ER272 reverts the NOD impairment and improves their capacity to perform in the MWM test, being part of the novelty of this work the identification of a compound that is able to revert spatial and episodic memory impairment.

The study of hippocampal neurogenesis in these mice shows that the generation of new hippocampal neurons is also compromised in this model of pathological aging. Previous results show a significant increase in proliferation in the DG of two‐month‐old SAMP8 animals compared with SAMR1 that returns to control levels in older mice (Diaz‐Moreno et al., 2013). Accordingly, we do not see any changes in the proliferation of cells (BrdU^+^) in the DG of six‐month‐old SAMP8 mice compared with SAMR1; however, we observe a robust increase in proliferation in SAMP8 mice that have been treated with ER272 from the 4th to the 6th month of age, indicating that the proliferative response initiated before 2 months of age is maintained in treated mice as a consequence of the treatment. Interestingly, in the 6th month, we observe a reduction in the number of neuroblasts (DCX^+^ cells) and in the number of DCX^+^ neuroblasts that incorporated BrdU over the course of the 2 months before sacrifice in SAMP8 mice compared with SAMR1. The treatment of mice with ER272 avoided this reduction indicating the capacity of this compound to potentiate the generation of neuroblasts.

It was quite noticeable the aberrant morphology of the DCX^+^ cells on the SAMP8 mice compared with the SAMR1 control group. These results complement previous ones, which show an abnormal accumulation of the DCX^+^ cell population in two‐month‐old SAMP8 mice. The location of DCX^+^ cells was altered, these cells migrated deeper into the granule cell layer and were aberrantly positioned (Diaz‐Moreno et al., 2018). We show here an aberrant morphology of DCX^+^ cells in the GCL (granular cell layer) characterized by shorter dendrites, a lower number of dendritic segments, and a general retraction of the dendritic tree in 6‐month‐old SAMP8 mice. However, the treatment of mice with ER272 prevents the development of these aberrant morphological alterations found in the aged mouse brain.

The reduction in the number of DCX^+^ neuroblasts in SAMP8 mice was not concomitant with a reduction in the activation of NSC (GFAP^+^SOX2^+^MCM2^+^ cells). In this context, an explanation for the lower number of DCX^+^ cells could be that we also detected an increase in the number of newly generated astrocytes S100β^+^BrdU^+^ suggesting that not all of the activated NSC are producing neuroblasts, but they are generating astrocytes. Previous reports have shown that astroglial differentiation of neural stem cells is a hallmark of neurogenesis in the aged brain also indicating that NSC is lost in aged mice due to their preferential differentiation into mature astrocytic cells (Diaz‐Moreno et al., 2018; Encinas et al., 2011). We observe here that indeed an elevated number of mature astrocytes can be observed in 6‐month‐old SAMP8 mice compared with SAMR1 and it is noticeable the increase in the number of astrocytes that have incorporated BrdU within the 8‐week period prior to sacrifice. Notwithstanding, the treatment of SAMP8 mice for 8 weeks prevented this phenomenon reducing the number of BrdU^+^ astrocytes to that found in SAMR1 mice and increasing the number of DCX^+^ neuroblasts.

Noteworthy was the fact that despite the reduction in DCX^+^ cells in the SAMP8 mouse compared with SAMR1, no differences were found in the number of NeuN^+^ neurons or NeuN^+^ neurons that incorporated BrdU over the course of the 2 months before sacrifice. These puzzling results agree with the previous reports that show in five‐month‐old SAMP8 that the number of NeuN^+^ cells that have incorporated BrdU (administered daily) over the last month of life is not different from that in SAMR1 mice (Sasaki et al., 2020). An explanation for the lack of differences in the number of neurons that have been generated in the SAMP8 is that the survival rate of newly generated neurons in the SAMP8 mice might be higher than in SAMR1 or that differentiation is accelerated as a response. Interestingly, the number of DCX^+^ cells that expressed NeuN was also reduced in SAMP8 mice compared with SAMR1, indicating that a higher rate of differentiation may not be the reason for the lack of differences in NeuN^+^BrdU^+^ cells and suggesting that in SAMP8 the survival rate of NeuN^+^ cells may be higher than in SAMR1. The treatment of SAMP8 mice with ER272 led to a higher number of DCX^+^ and NeuN^+^ cells that had incorporated BrdU and a higher number of DCX^+^NeuN^+^ cells' life indicating that this pharmacological agent was positively regulating neurogenesis by facilitating differentiation and probably not affecting survival.

Previous studies show that the proportion of NSC decreases with age in the mouse brain reducing their senescent cells' ability to undergo activation and remaining inactive or quiescent for prolonged periods (Diaz‐Moreno et al., 2018; Harris et al., 2021; Ibrayeva et al., 2021; Martin‐Suarez et al., 2019), thus preserving the NSC reservoir from exhaustion. However, for the continuous generation of new neurons, a certain basal firing rate is required (Ziebell et al., 2018). We have found here that in the DG of SAMP8 mice, a reduction in the number of NSC is observed with age, as demonstrated by the lower number of radial glial‐like cells that express SOX2 and GFAP. This reduction is concomitant with an increase in astrogliosis. A similar depletion of NSC has previously been observed in the DG of six‐month‐old SAMP8 mice compared with SAMR1 mice accompanied by a response in NSC activation (Diaz‐Moreno et al., 2018). Using our experimental model this activation is slightly higher, but this increase does not reach statistical significance. It was clear, though, that the treatment of mice with ER272 increased the number of activated NSC compared with nontreated SAMP8 mice and with SAMR1, thus indicating that this treatment activates NSC and reduces the NSC pool. We cannot discard the possibility that prolonged treatment with this compound might drain the NSC pool in the long‐term. The maintenance of quiescence is probably a determining factor when it comes to preserving the neurogenic rate during aging because it protects the NSC reservoir from total exhaustion (Encinas & Sierra, 2012). However, a certain basal activation rate is required (Ziebell et al., 2018) that allows the generation of new neurons to maintain cognitive performance. These results suggest that the search for compounds that act on other events of the neurogenic hierarchical process may be an interesting alternative to promote neurogenesis in the aging brain (i.e., the proliferation of progenitors, their differentiation into neuroblasts, or the survival and maturation of newly generated neurons).

Finally, in order to understand the mechanisms of action that lead to the effect of ER272, we have analyzed its role in the release of TGFα to the extracellular medium. TGFα is one of the physiological ligands of the EGFR, and as other EGFR ligands, it is expressed as a membrane‐bound proligand with the EGFR‐binding domain in the extracellular N‐terminal portion of the protein. The shedding of the EGFR binding domain that releases the ligand is catalyzed by a metalloprotease of the ADAM family, ADAM17, via the regulation of protein kinase C (Dang et al., 2011, 2013). Using a fusion protein construct in which pro‐TGFα was cloned in frame with an eGFP protein in the C‐terminal end and a mCherry protein in the N‐terminal, we show that in culture cells, ER272 facilitates the release of TGFα as shown in previous reports (Dominguez‐Garcia et al., 2020). This release is impaired in cells in which the expression of classical protein kinase C alpha (PKCα) has been abolished, indicating that ER272 stimulated the PKCα dependent release of TGFα. Accordingly, in SAMP8 mice treated with ER272 for 2 months, we observe an increase in the concentration of TGFα in the CSF whereas we do not observe alterations on TGFα expression in any of the experimental groups indicating that the treatment may be acting on the release not altering the expression pattern. This increase is concomitant with the stimulation of the MAPK/ERK1/2 signaling cascade. The activation of this pathway is reduced in SAMP8 mice and the treatment with ER272 restores this activity. This finding agrees with a previous report in which SAMP8 mice show a reduced pERK1/2 activity that is related to changes in cognitive performance and neuronal morphology (Vasilopoulou et al., 2022). The implication of TGFα in cognitive performance has previously been suggested agreeing with our findings (Alipanahzadeh et al., 2014; Vasilopoulou et al., 2022). Concerning its origin, TGFα is expressed in the hippocampus of several mammalian species (Lazar & Blum, 1992) participating in the postpuberal formation of brain structures (Koshibu et al., 2005; Koshibu & Levitt, 2005). In the hippocampus, TGFα is found at higher levels than other EGFR ligands such as the epidermal growth factor (Kaser et al., 1992). This signaling molecule may have a neuronal or glial origin and participates in neural progenitor proliferation/cell fate choice or neuronal survival/differentiation (Junier, 2000). Although our results suggest that an increase in TGFα may be one of the mechanisms involved in the positive regulation of neurogenesis found in SAMP8 mice avoiding cognitive impairment, we cannot discard the possibility of other signaling molecules being involved in the effect of ER272. We had previously shown that ER272 lightly stimulates the release of neuregulin 1 (Dominguez‐Garcia et al., 2020). Since neuregulin stimulates adult hippocampal neurogenesis, we cannot discard the possibility of this molecule being responsible for part of the observed effect.

In light of the obtained results, it must be mentioned that other phorbols like phorbol myristate acetate (PMA) have shown tumor‐promoting activities. We have selected the use of ER272 because of the similarities in structure with the non‐tumor‐promoting diterpene prostratin (Geribaldi‐Doldan et al., 2019). We do not believe that diterpene ER272 may promote tumor development because its capacity to promote proliferation in brain tumors was tested previously together with other prostratin‐like 12‐deoxyphorbols (Geribaldi‐Doldan et al., 2015). We did not observe any tumor in any of the mice used in the experiment. Notwithstanding, the effect of ER272 on glioblastoma and other brain tumor development would need to be further tested.

In summary, several pieces of evidence show the existence of alterations in neurogenesis in aged mice (Diaz‐Moreno et al., 2018; Kempermann et al., 1998; van Praag et al., 2005), some of them suggesting that neurogenesis does not occur in the neuropathologically aged DG as it does in the nonaged niche. As previously reported, we have observed that neurogenesis (Diaz‐Moreno et al., 2018) and cognitive performance (Dobarro et al., 2013; Takeda, 2009) is impaired in the brain of a mouse model of pathological aging. Nevertheless, we report the effect of the diterpene with 12‐deoxyphorbol structure ER272, a pharmacological compound that positively regulates adult hippocampal neurogenesis by increasing the proportion of activated NSC, favoring the generation of neuroblasts, neuronal differentiation, and reorganizing neurogenesis. Thus, our results suggest that this compound helps rejuvenating the DG niche. Concomitantly, we show an effect of this drug improving cognitive performance in the same model. The study of the mechanism of action reveals that an increase in TGFα release via the activation of PKCα may be the underlying mechanism of action. It could then be concluded that we have found a small molecule that may work as a pharmacological drug to positively regulate neurogenesis and cognitive performance in the pathologically aged brain.

## AUTHOR CONTRIBUTIONS

Ricardo Gómez‐Oliva, Pedro Nunez‐Abades, and Monica Garcia‐Alloza involved in experimental design, data acquisition and analysis, discussion of results, article preparation, and writing. Sergio Martínez‐Ortega, Isabel Atienza‐Navarro, Carlos Bernal, Samuel Domínguez‐García, Noelia Geribaldi‐Doldán, and Cristina Verástegui involved in data acquisition and analysis, and discussion of results. Rosario Hernández‐Galán and Abdellah Ezzanad involved in isolation characterization and preparation of chemical compound. Carmen Castro involved in conception of the work, experimental design, data acquisition and analysis, discussion of results, article preparation and writing, and funding acquisition.

## FUNDING INFORMATION

This work was supported by the Spanish Agencia Estatal de Investigación (grant number RTI‐2018‐099908‐B‐C21 and RTI‐2018‐099908‐B‐C22 granted to Carmen Castro). This work has been co‐financed by the European Union under the 2014‐2020 ERDF Operational Programme and by the Department of Economic Transformation, Industry, Knowledge, and Universities of the Regional Government of Andalusia. Project reference: (grant number FEDER‐UCA18‐106647 granted to Carmen Castro). This work has been co‐financed and by the Consejería de Salud y Familias and by EDRF ITI regional funds (80%) (Grant number: ITI‐Cadiz‐0042‐2019 granted to Carmen Castro).

## CONFLICT OF INTEREST STATEMENT

The authors declare that the research was conducted in the absence of any commercial or financial relationships that could be construed as a potential conflict of interest.

## Supporting information


Data S1

Movie S1

## Data Availability

Data will be available to interested person upon request.
