# Supplementary material for: Rescue of neurogenesis and age‐associated cognitive decline in SAMP8 mouse: Role of transforming growth factor‐alpha
Source: Aging Cell. 2023 May 12;22(6):e13829. doi: 10.1111/acel.13829 (PMC10265154; doi:10.1111/acel.13829)
Supplement: Supplementary file 1 — Data S1 [file ACEL-22-e13829-s001.pdf]

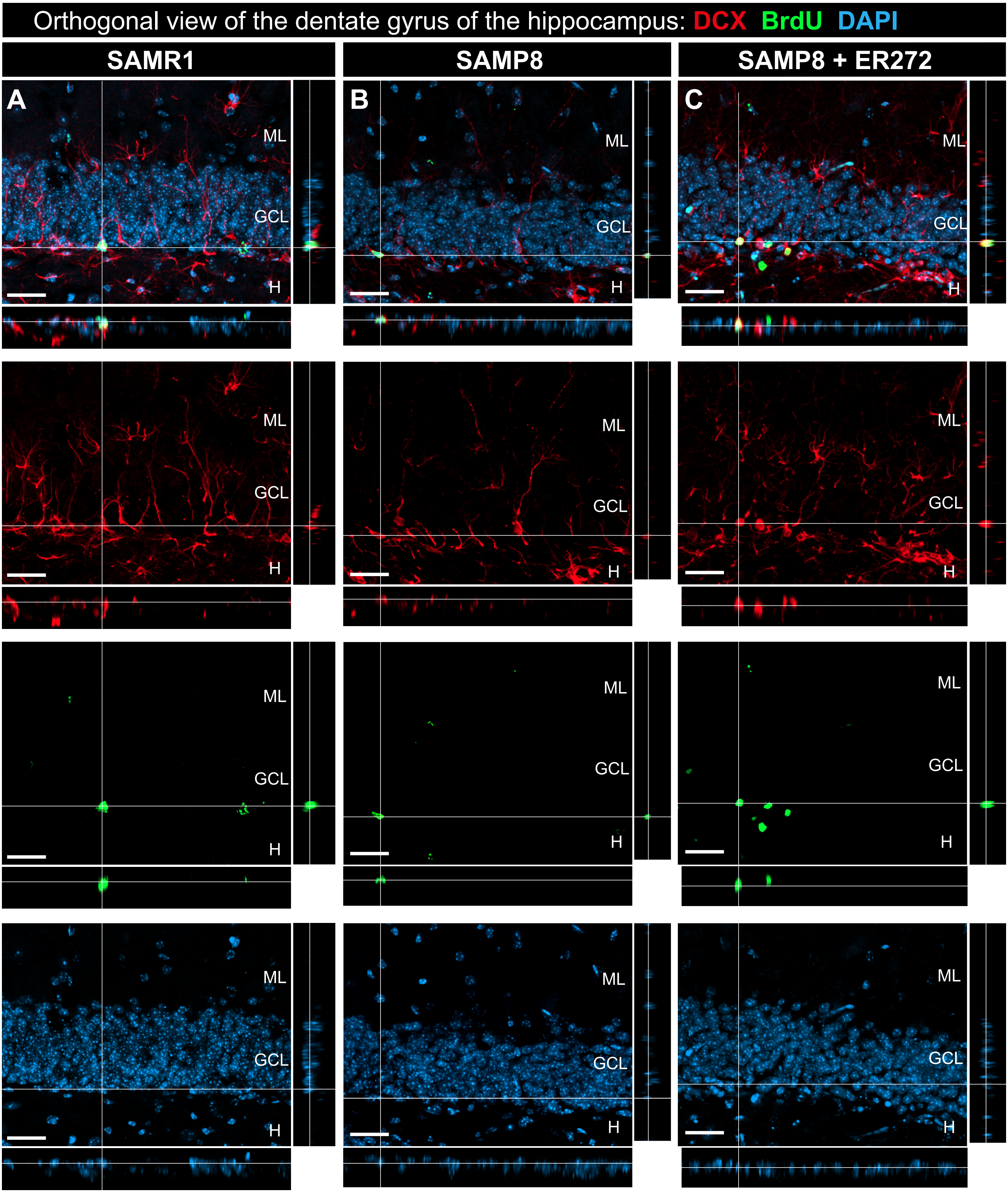

Supplementary Figure S1. Effect of long-term intranasal administration of ER272 to SAMP8 mice on DCX⁺BrdU⁺DAPI⁺ cells. A-C) Representative orthogonal view images of the DG of the hippocampus of six-month-old SAMR1 and SAMP8 male mice treated with vehicle (A, B respectively) or SAMP8 male mice treated with ER272 (C) during eight weeks as indicated in Fig. 1 A. Slices were processed for the immunohistochemical detection of the proliferation marker BrdU (lower medium panel; green) and DCX (upper medium panel; red). DAPI staining is shown in blue (lower panel). Merged channels are shown in the upper panel. Scale bar represents 25 μm.

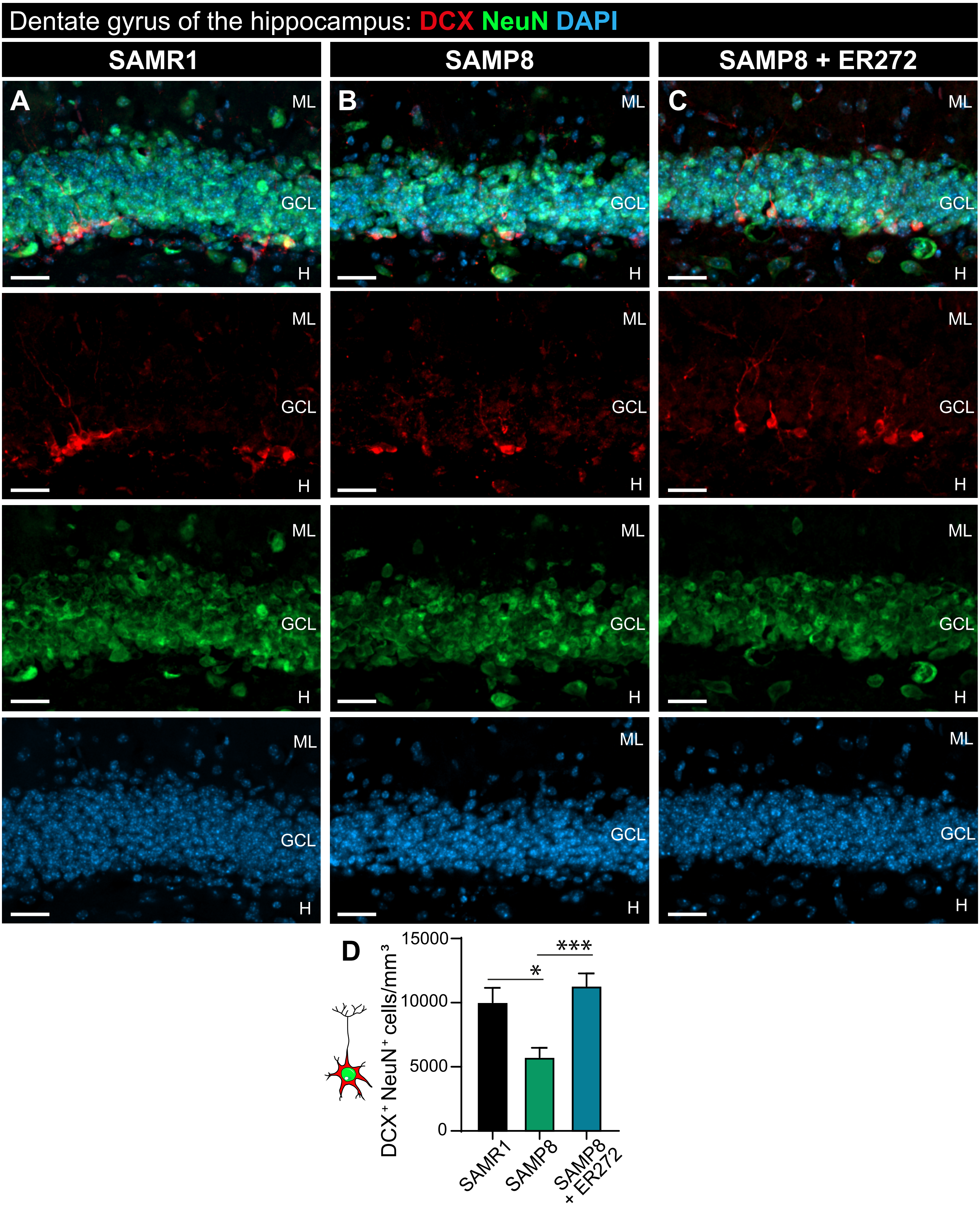

Supplementary Figure S2. Effect of long-term intranasal administration of ER272 to SAMP8 mice on DCX⁺NeuN⁺ DAPI⁺ cells. A-C) Representative confocal microcopy images of the DG of the hippocampus of six-month-old SAMR1 and SAMP8 male mice treated with vehicle (A, B respectively) or SAMP8 mice treated with ER272 (C) during eight weeks as indicated in Fig. 1 A. Slices were processed for the immunohistochemical detection of DCX (upper medium panel; red) and NeuN (lower medium panel; green) markers. DAPI staining is shown in blue (lower panel). Merge channels are shown in the upper panel. **D)** Graph shows the number of DCX⁺NeuN⁺ cells in the DG of the hippocampus per mm^3 [$F_{(2,30)}$=8.977, *$p<0.011$ SAMR1 vs SAMP8] [$F_{(2,30)}$=8.977, ***$p<0.001$ SAMP8 vs SAMP8+ER272]. Data are the means ± S.E.M of six animals, n=6. Differences detected by one-way ANOVA followed by Tukey b test. Scale bar represents 25 μm.

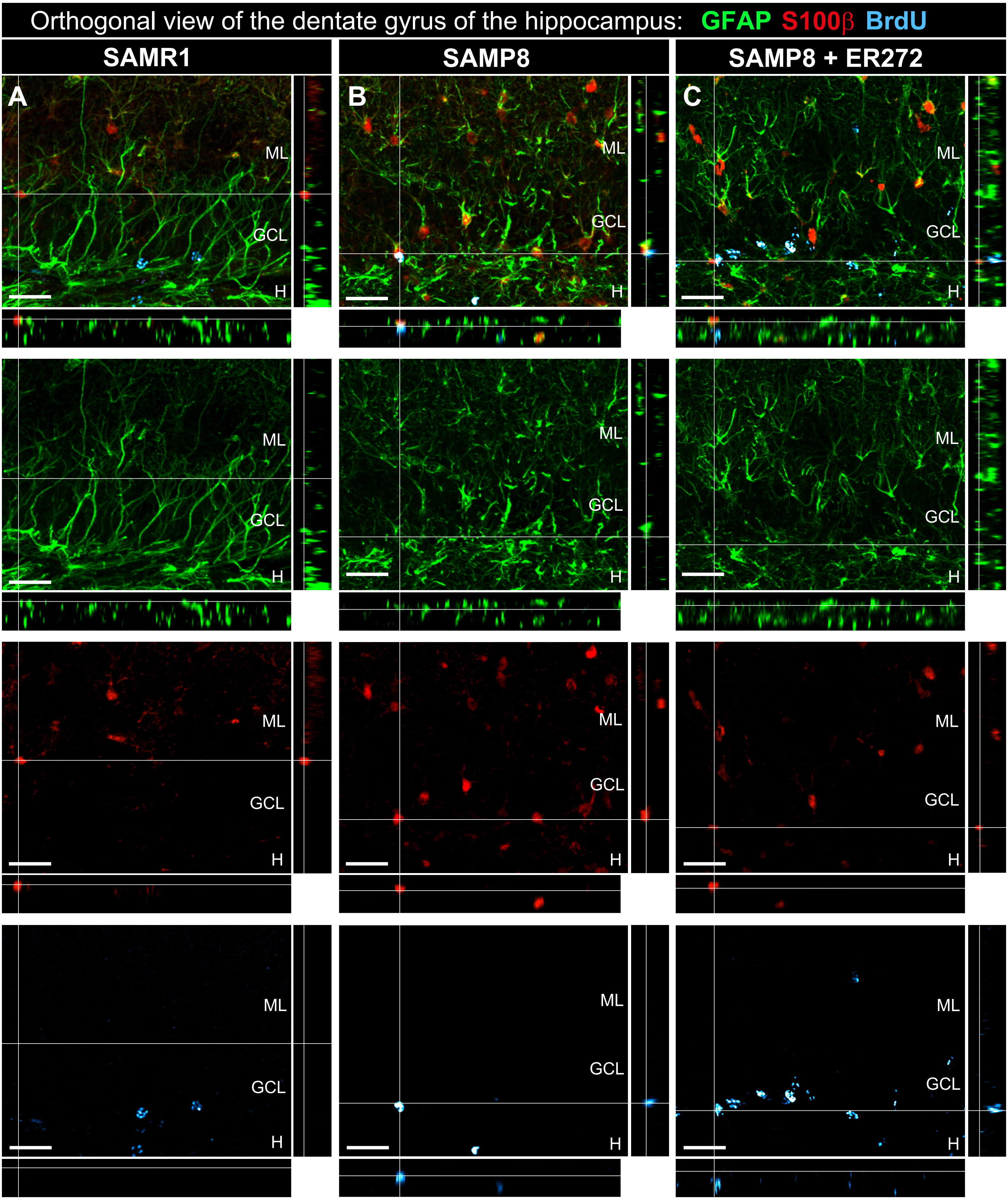

Supplementary Figure S3. Effect of long-term intranasal administration of ER272 to SAMP8 mice on GFAP⁺S100β⁺ BrdU⁺ cells. A-C) Representative orthogonal view images of the DG of the hippocampus of six-month-old SAMR1 and SAMP8 male mice treated with vehicle (A, B respectively) or SAMP8 mice treated with ER272 (C) during eight weeks as indicated in Fig. 1 A. Slices were processed for the immunohistochemical detection of the Glial Fibrillary Acidic Protein, GFAP (upper medium panel; green); S100β (lower medium panel; red); and BrdU (lower panel; cyan). Merged channels are shown in the upper panel. Scale bar represents 25 μm.

Gómez-Oliva et al. Supplementary Figure S4

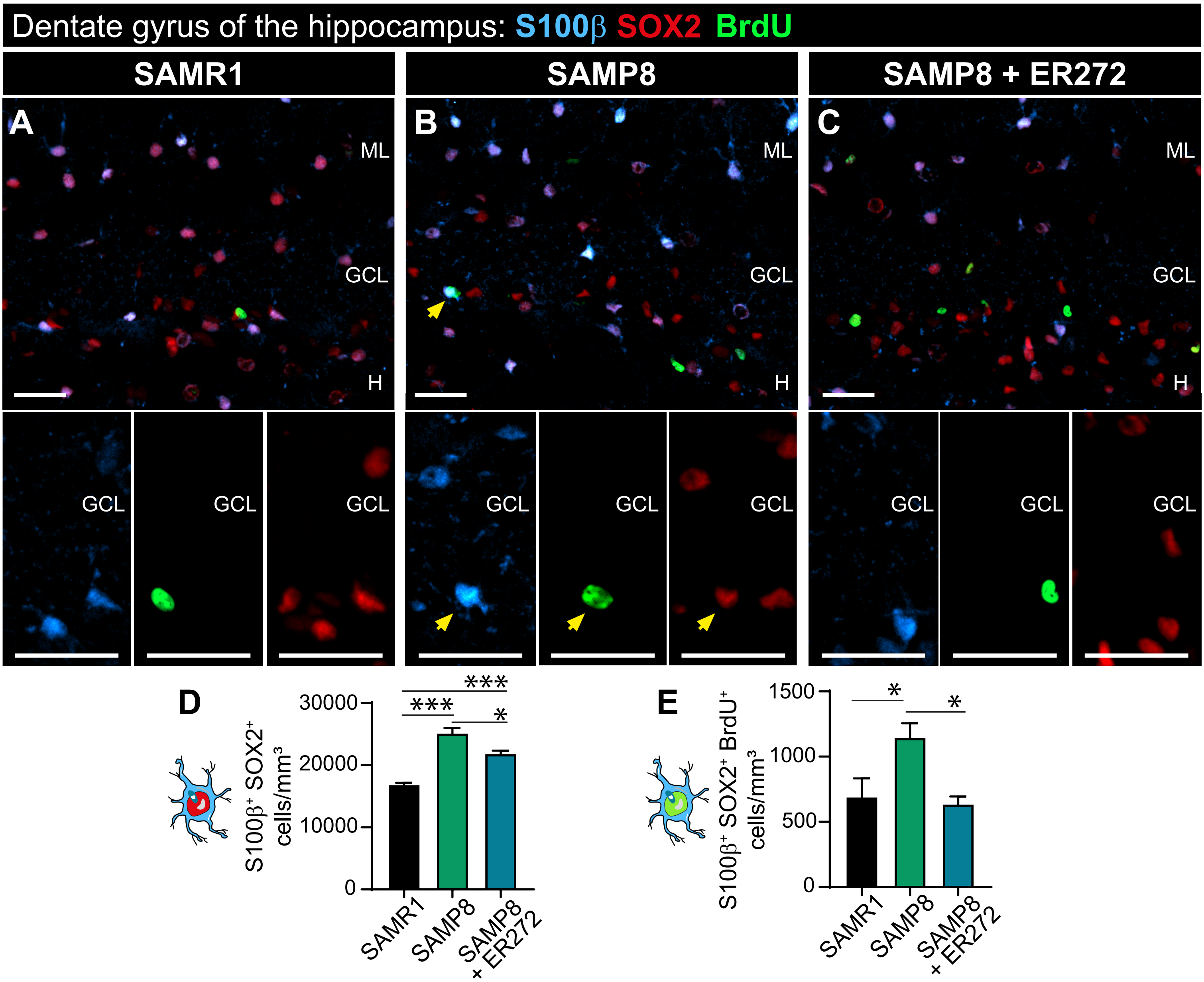

Supplementary Figure S4. Intranasal administration of ER272 decreases the number of newly generated astrocytes in the dentate gyrus of SAMP8 mice. A-C) Representative confocal microcopy images of the DG of the hippocampus of six-month-old SAMR1 and SAMP8 male mice treated with vehicle (A, B respectively) or SAMP8 mice treated with ER272 (C) during eight weeks as indicated in Fig. 1 A. Slices were processed for the immunohistochemical detection of the proliferation marker BrdU (green), SOX2, a transcription factor in astrocytes (red) and the marker for astrocytes S100β (cyan). Yellow arroows indicate S100β$^+$SOX2$^+$BrdU$^+$ cells. **D)** Graph shows the number of S100β$^+$SOX$^+$ cells in the DG per mm^3 [$F_{(2,13)}$=46.0,***p<0.01 SAMR1 vs SAMP8] [$F_{(2,13)}$=46.0,***p<0.01 SAMR1 vs SAMP8+ER272] [$F_{(2,13)}$=46.0,*p=0.013 SAMP8 vs SAMP8+ER272]. **E)** Graph shows the number of S100β$^+$SOX2$^+$BrdU$^+$ cells in the DG of hippocampus per mm^3 [$F_{(2,13)}$=5.48, *p<0.036 SAMR1 vs SAMP8] [$F_{(2,13)}$=5.48, *p=0.036 SAMP8 vs SAMP8+ER272]. Data are the means ± S.E.M of six animals, n=6. Differences detected by one-way ANOVA followed by Tukey b test. Scale bar represents 25 µm.

Gómez-Oliva et al. Supplementary Figure S5

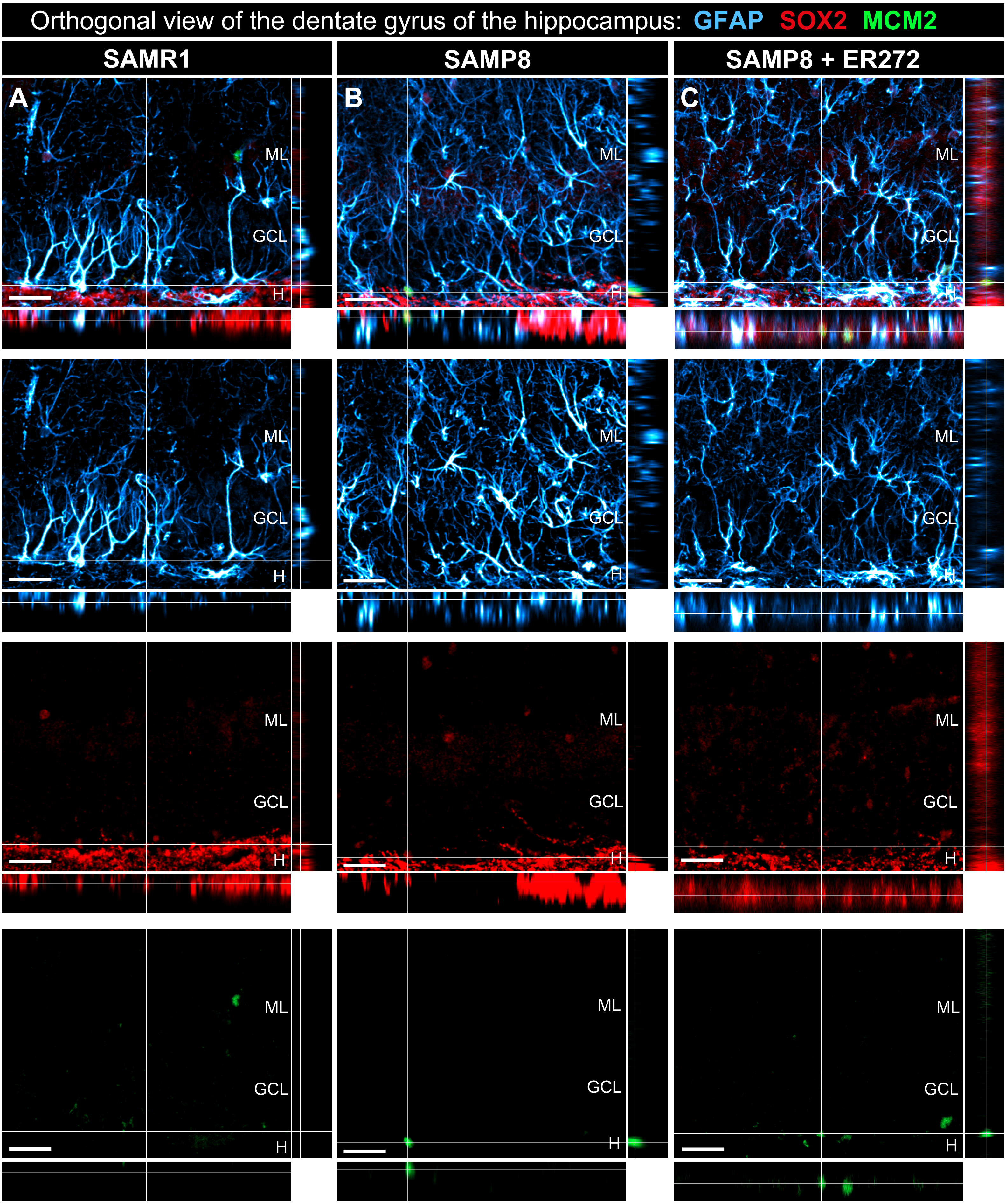

Supplementary Figure S5. Effect of long-term intranasal administration of ER272 to SAMP8 mice on GFAP^{+}SOX2^{+} MCM2^{+} cells. A-C) Representative orthogonal view of the DG of the hippocampus of six-month-old SAMR1 and SAMP8 male mice treated with vehicle (A, B respectively) or SAMP8 mice treated with ER272 (C) during eight weeks as indicated in Fig. 1A. Slices were processed for the immunohistochemical detection of the Glial Fibrillary Acidic Protein, GFAP (cyan); SOX2 (red); and the cell cycle marker MCM2 (green). Scale bar represents 25 μm.

Gómez-Oliva et al. Supplementary Figure S6

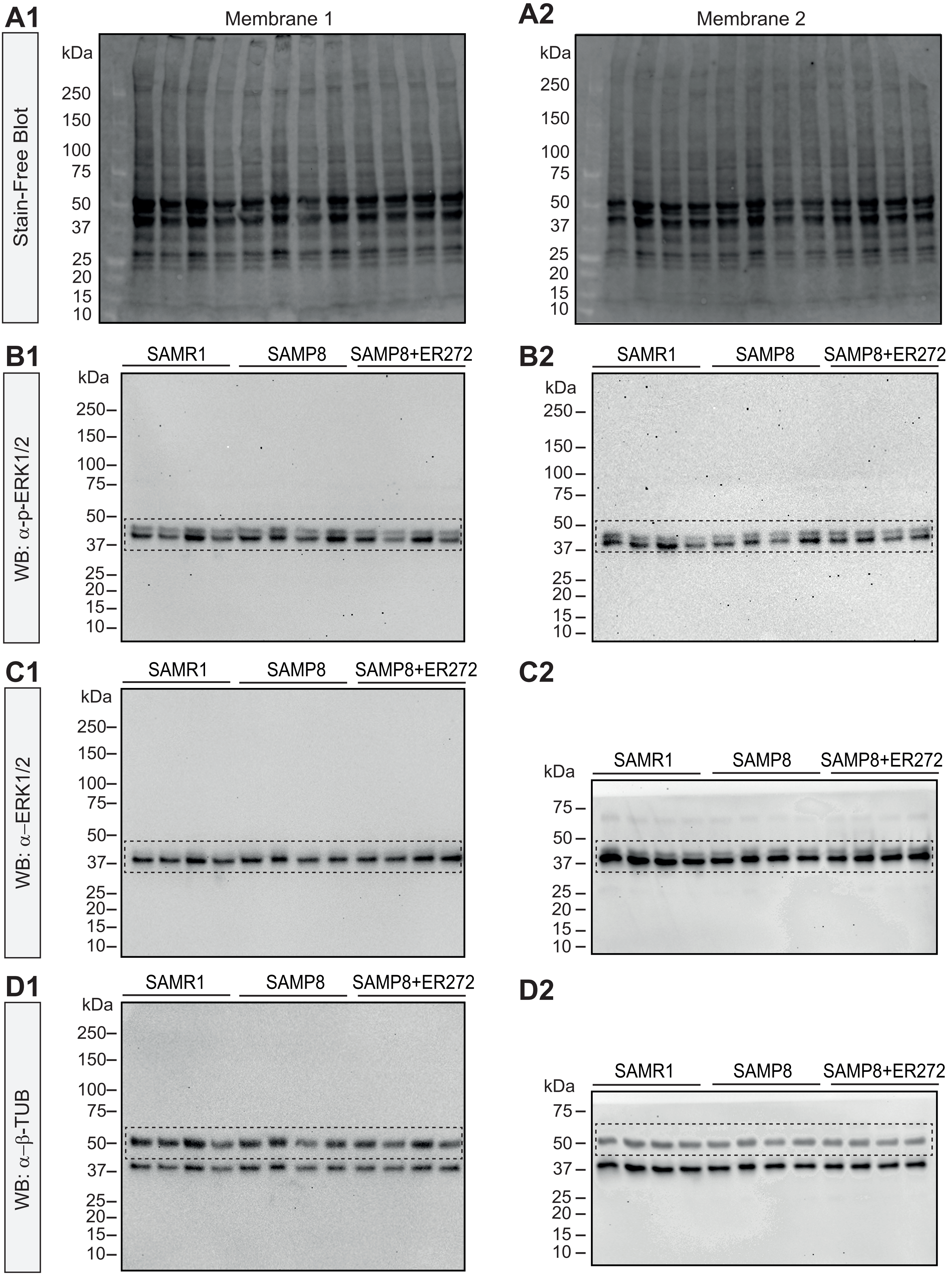

Supplementary Figure S6. Total hippocampal protein membrane imaging and antibody detection. A) Total hippocampal proteins of six-month SAMR1 and SAMP8 male mice treated with vehicule or SAMP8 mice treated with ER272, separated on 4-15% polyacrylamide gels and transferred to PVDF membranes (membrane 1 and membrane 2). **B)** Images of chemiluminescence signal of immunoblot detection of p-ERK1/2 (dotted squares). B2 image conrresponds to representative blots in the main figure. **C)** Images of chemiluminescence signal of immunoblot detection of total ERK1/2 (dotted squares). C2 image conrresponds to representative blots in the main figure. **D)** Images of chemiluminescence signal of immunoblot detection of loading control β-tubulin (dotted squares). D2 image conrresponds to representative blots in the main figure. n=8.

Antibody	Host	Isotype	Epitope retrieval	Staining pattern	Source	Reference
Anti-DCX	Rabbit	Polyclonal	DCX, neuroblast marker	Cytoplasmic	Abcam (Cambridge, UK)	ab18723
Anti-S100β	Rabbit	Polyclonal	S100β, astrocyte marker	Cytoplasmic	Abcam (Cambridge, UK)	ab41548
Anti-NeuN	Rabbit	Monoclonal	NeuN, neuronal marker	Nuclear	Abcam (Cambridge, UK)	ab177487
Anti-phospho-p44/42 MAPK (ERK1/2)	Rabbit	Polyclonal	Phospho-p44/42 MAPK (ERK1/2), marker of stimulation of MAPK (ERK1/2) signaling cascade	Cytoplasmic/ nuclear	Cell Signaling (Danvers, MA, USA)	9101
Anti-SOX2	Rabbit	Polyclonal	SOX2, astrocyte/stem cell marker	Nuclear	Santa Cruz Biotechnology, Santa Cruz, CA, USA)	sc-20088
Anti-SOX2	Goat	Polyclonal		Nuclear	RD Systems Minneapolis, CO, USA)	AF2018
Anti-MCM2	Mouse	Monoclonal	MCM2, cell proliferation marker	Nuclear	BD Bioscience (East Rutherford, NJ, USA)	610701
Anti-p44/42 MAPK (ERK1/2)	Mouse	Monoclonal	p44/42 MAPK (ERK1/2), marker of total MAPK (ERK1/2)	Cytoplasmic/ nuclear	Cell Signaling (Danvers, MA, USA)	4696
Anti-β-tubulin	Mouse	Monoclonal	β-tubulin, core protein in microtubules, loading control for Western Blot	Cytoplasmic	Thermo Fisher Scientific (Waltham, MA, USA)	MA5-16308-HRP
Anti-GFAP	Chicken	Polyclonal	GFAP, glial marker	Cytoplasmic	Abcam (Cambridge, UK)	ab4674
Anti-NeuN	Rat	Monoclonal	NeuN, neuronal marker	Nuclear	Abcam (Cambridge, UK)	ab279297
Anti-BrdU	Rat	Monoclonal	BrdU, cell proliferation marker	Nuclear	Abcam (Cambridge, UK)	ab6362

Primary antibodies supplementary table 1: List of primary antibodies used in the study. Specifying host, isotype, epitope retrieval, staining pattern, source and reference.

Antibody	Host	Dilution	Fluorescence	Source	Reference
Alexa Flour anti-rabbit	Donkey	1:1000	647	Invitrogen (Carlsbad, CA, USA)	A-32795
Alexa Flour anti-rabbit	Donkey	1:1000	594	Invitrogen (Carlsbad, CA, USA)	A-21207
Alexa Flour anti-rabbit	Donkey	1:1000	488	Invitrogen (Carlsbad, CA, USA)	A-21206
Alexa Flour anti-goat	Donkey	1:1000	647	Invitrogen (Carlsbad, CA, USA)	A-11058
Alexa Flour anti-mouse	Donkey	1:1000	488	Invitrogen (Carlsbad, CA, USA)	A-21206
Alexa Flour anti-chicken	Goat	1:1000	647	Abcam (Cambridge, UK)	ab150175
Alexa Flour anti-rat	Donkey	1:1000	488	Life Technologies (Eugene, OR, USA)	A-21208

Secondary antibodies supplementary table 2: List of secondary antibodies used in the study. Specifying host, dilution used, fluorescence conjugated, source and reference.

Intercalating agent	Target	Staining pattern	Source	Reference
DAPI	Double-stranded DNA binding dye	nuclear	(Sigma, St. Louis, MO, USA)	D9542

DNA staining: DNA-intercalanting-dye used to stain cell nuclei. Specifying target, staining pattern, source and reference.
